# Inhibition of hypoxic exosomal miR-423-3p decreases glioma progression by restricting autophagy in astrocytes

**DOI:** 10.1038/s41419-025-07576-2

**Published:** 2025-04-08

**Authors:** Ziyi Tang, Zhiwei Xue, Xuchen Liu, Yan Zhang, Jiangli Zhao, Junzhi Liu, Lin Zhang, Qindong Guo, Bowen Feng, Jiwei Wang, Di Zhang, Xingang Li

**Affiliations:** 1 Department of Neurosurgery, Qilu Hospital, Cheeloo College of Medicine and Institute of Brain and Brain-Inspired Science, Shandong University, Jinan, China; 2 Jinan Microecological Biomedicine Shandong Laboratory and Shandong Key Laboratory of Brain Health and Function Remodeling, Jinan, China; 3 Department of Clinical Laboratory, Qilu Hospital of Shandong University, Jinan, Shandong China

**Keywords:** Cancer microenvironment, Cancer in the nervous system, miRNAs

## Abstract

The tumor microenvironment (TME) of gliomas comprises glioma cells and surrounding cells, such as astrocytes, macrophages, T cells, and neurons. In the TME, glioma cells can activate normal human astrocytes (NHAs) through the secretion of exosomes and the activation of astrocytes can further improve the progression of glioma, leading to a poor prognosis for patients. However, the molecular mechanisms underlying NHAs activation by gliomas remain largely unknown. It this study, glioma-derived exosomes (GDEs) play an important role in the modulation of autophagy and activation of NHAs. Compared with normoxic GDEs, hypoxic glioma-derived exosomes (H-GDEs) further improved autophagy and activation of astrocytes, which strongly promoted the progression of glioma cells. In an miRNA array between two types of exosomes from gliomas, miR-423-3p was highly expressed in H-GDEs and played an important role in autophagy, resulting in the activation of NHAs. The mechanism by which hypoxic glioma cells react with NHAs to create an immunosuppressive microenvironment was identified and 15d-PGJ2 was established as an effective inhibitor of miR-423-3p to suppress NHAs activation. These findings provide new insights into the diagnosis and treatment of gliomas by targeting autophagy and miR-423-3p expression.

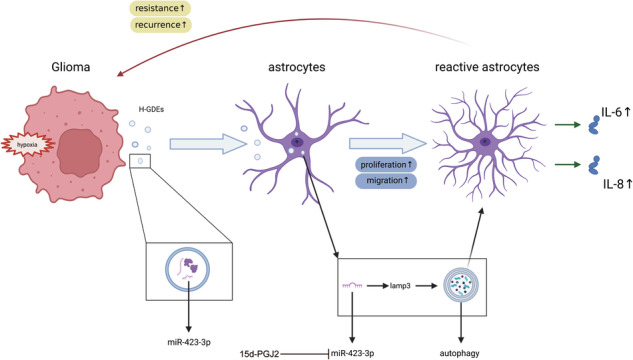

## Introduction

Glioma is the most malignant and aggressive type of primary brain tumor, with a median survival of 12–15 months in adults [1, 2]. Despite recent advances in therapeutic strategies for glioblastoma multiforme (GBM), incorporating multimodal techniques, such as surgery, chemotherapy, and radiotherapy, the treatment of glioma is still very limited owing to its aggressive invasiveness and high recurrence rate [3]. Formation of an immunosuppressive microenvironment composed of endothelial cells, neurons, and astrocytes is largely influenced by glioma cells. Divergent cells within the tumor microenvironment (TME) synchronize to increase the proliferation, invasion, immune suppression, and angiogenesis of glioma cells [4, 5]. Therefore, interfering with the crosstalk between tumor cells and TME has become a novel strategy to address the growth and recurrence of gliomas.

Hypoxia caused by mismatched growth between blood vessels and the glioma volume is considered responsible for the malignant behavior of gliomas [6]. Hypoxia increases the resistance [7], stemness, invasion, and angiogenesis of gliomas [8]. Under hypoxic conditions, similar to the influence of irradiation and temozolomide, hypoxia can induce normal astrocytes to become reactive astrocytes (RAs) [9], indicating that hypoxia is an important factor in promoting TME progression.

Astrocytes are the most abundant type of glial cell in the brain and play an important role in the maintenance of neuronal function. The ability of astrocytes to proliferate extensively in response to injuries and insults has led to the hypothesis that astrocytes could serve as the cells of origin for gliomas. Astrocytes can be differentiated into tumor glial cells or tumor-origin cells by overexpressing oncogenes in vivo and in vitro [4]. In addition, RAs can induce the release of O6-alkylguanine DNA alkyltransferase mRNA from cells via exosomes. These exosomes can be absorbed into glioma cells by endocytosis to block temozolomide (TMZ)-induced apoptosis and improve their TMZ resistance [10, 11]. Accumulating evidence has confirmed that RAs promote glioma progression; however, whether gliomas contribute to astrocyte activation remains unclear.

Exosomes are cell-secreted, vesicle-like corpuscles 30–120 nm in diameter. These vesicles deliver various components, such as proteins and RNA, to target cells via fluid circulation. Tumor cells can communicate with distant cell types by secreting exosomes to transfer genetic material and create an immunosuppressive TME [12, 13]. Under hypoxic conditions, tumors increase their content and enhance the secretion of exosomes, which subsequently exert detrimental effects on recipient cells [14–16]. Notably, RAs can release exosomes into glioma cells and increase their chemical resistance [10]. Therefore, exosomes play an important role in mediating the crosstalk between gliomas and astrocytes.

MicroRNAs (miRNAs) are endogenous small RNAs (20–24 nucleotides) [17]. These molecules have many regulatory effects on cells, and beneficial effects on glioma proliferation and progression [3, 18]. In addition, miRNAs induce M2-like polarization in peritumoral macrophages, indicating their important role in the TME.

Autophagy is a process in which lysosomes phagocytose cytoplasmic proteins and organelles to form autophagosomes, which then degrade their contents [19]. Autophagy can protect cells from death under adverse circumstances, such as hypoxia, starvation, and chemotherapy [20]. Despite its beneficial role under physiological conditions, dysregulation of autophagy promotes the proliferation and growth of tumors. In pancreatic ductal adenocarcinoma, tumor cells specifically promote autophagy in pancreatic stellate cells (PSCs) and induce the degradation of proteins in PSCs to produce alanine, which can be taken up by tumor cells to promote their metabolic activity [21]. Furthermore, in gliomas, tumor cells can induce M2 macrophage polarization via enhanced autophagy [22]. Therefore, tumor cells can induce autophagic dysregulation in the TME to facilitate their growth.

15-deoxy-(Delta12,14)-prostaglandin J2 (15d-PGJ_2_) is a selective peroxisome proliferator-activated receptor gamma (PPARγ) agonist and a covalent peroxisome proliferator-activated receptor δ (PPARδ) agonist. It inhibits tumor growth through its pro-apoptotic and anti-angiogenic effects. In glioma, 15d-PGJ_2_ induces A172 glioma cell death by increasing reactive oxygen species (ROS) and causing mitochondrial dysfunction [23], highlighting its potential in glioma treatment. Additionally, in the TME, 15d-PGJ_2_ suppresses angiopoietin-II and inflammatory cytokines (IL-1β, TNF-α, and TGF-β) by activating PPARγ and producing ROS, thereby modulating tumor vasculature [24]. Therefore, 15d-PGJ_2_ may also influence astrocyte activation in the glioma microenvironment, though the underlying mechanisms require further investigation.

In this study, whether GDEs contribute to the activation of normal astrocytes in the TME was investigated and the underlying mechanisms were explored. GDEs induce autophagy and activate normal human astrocytes (NHAs). Compared with GDEs under normal conditions (N-GDEs), hypoxic GDEs (H-GDEs) induce stronger activation of NHAs. Next, upregulated miRNAs in H-GDEs were compared to N-GDEs and found that only miR-423-3p in H-GDEs markedly induced autophagy in NHAs and subsequently promoted their activation. Moreover, the downstream cellular mechanisms were explored and LAMP3 was identified as an important molecule that mediates miR-423-3p-induced autophagy in NHAs. Using the Connectivity Map (CMap) database, 15-deoxy-Δ12,14-prostaglandin J2, a selective and covalent PPARδ agonist, was identified as a potent inhibitor capable of suppressing the activation of NHAs.

## Materials and methods

### Cell lines and culture

The glioma cell line, U251, was purchased from the Chinese Academy of Sciences Cell Bank. NHAs and the primary human GBM biopsy-propagated tumor cell line, P3#GBM, were kindly provided by Department of Biomedicine, University of Bergen, Norway. NHAs and U251 were cultured in Dulbecco’s modified Eagle’s medium (Thermo Fisher Scientific, Waltham, MA, USA) supplemented with 10% fetal bovine serum (FBS, Vivacell, Shanghai, China). P3#GBM cells were cultured in neurobasal medium (NBM, Gibco™, Waltham, MA, USA) with 2% B27 supplement (Thermo Fisher Scientific), 1% GlutaMAX (Thermo Fisher Scientific), 1/5000 recombinant human epidermal growth factor (Novoprotein, Shanghai, China), and 1/5000 recombinant human fibroblast growth factor 2 (Novoprotein).

### Exosome isolation

U251 cells were cultured in DMEM with 10% exosome-free FBS and P3#GBM cells were cultured in NBM as described above. Both cell types were cultured under normoxic (21% O_2_) and hypoxic (1% O_2_) conditions in a CB60 incubator (Binder GmbH, Tuttlingen, Germany). The supernatant was collected every 2 days, and exosomes from cells and blood were isolated using gradient centrifugation (2000 × *g*, 10 min; 5000 × *g*, 10 min) and ultracentrifugation (100,000 × *g*, 1 h) [25]. The collected exosomes were stored at −80 °C. The concentration of exosomes was determined using a BCA Protein Assay kit (Beyotime, Shanghai, China) according to the manufacturer’s instructions and the concentration of 0.1 μg/ml [19, 26, 27] was used to treat the NHAs.

Other materials and methods used in this study are provided in the Supplementary Materials.

## Results

### H-GDEs induce autophagy in NHAs in vitro

**Fig. 1:**
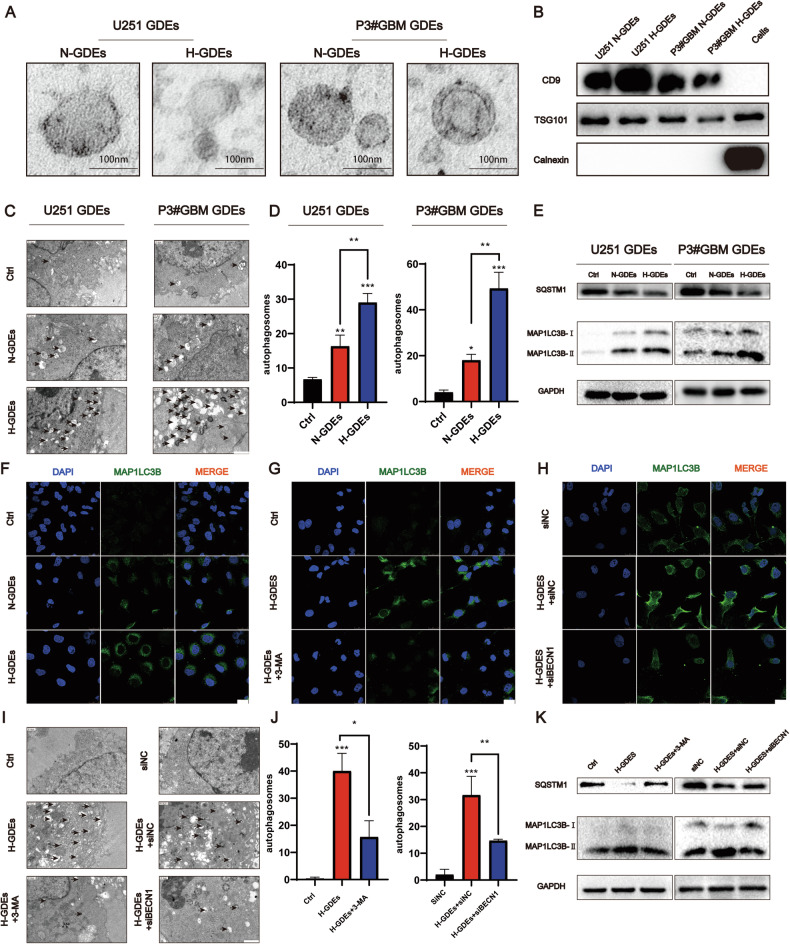
H-GDEs induce autophagy in NHAs in vitro. **A** TEM images of exosomes derived from U251 and P3#GBM under normoxia and hypoxia, respectively (scale bar, 100 μm). **B** Western blotting was used to detect the expression of CD9, tsg101, and calnexin in protein derived from P3#GBM, N-GDEs, and H-GDEs. Exosomes were extracted from U251 and P3#GBM under normoxia and hypoxia, respectively. **C**, **D** TEM Images Illustrating autophagosome formation in NHAs treated with PBS, N-GDEs, and H-GDEs from U251 and P3#GBM cells for 48 h. Autophagosomes are indicated by arrowheads. Representative images and statistical data on autophagosome counts (scale bar, 1.2 μm). **E** Western blotting was used to detect the expression levels of SQSTM1-P62, MAP1LC3B, and GAPDH in NHAs treated with PBS, N-GDEs, and H-GDEs derived from U251 and P3#GBM cells for 48 h. **F**–**H** Immunofluorescence staining for MAP1LC3B (green) to measure the level of autophagy in NHAs treated with PBS, N-GDEs, and H-GDEs derived from P3#GBM for 48 h; 3-MA and siBecn1 were added to the H-GDEs to inhibit autophagy and cell nuclei were stained with DAPI (blue). Representative images of three sets of experiments (scale bar, 25 μm). **I**, **J** TEM images illustrating autophagosome formation in NHAs treated with PBS, siNC, and H-GDEs derived from P3#GBM cells for 48 h; 3-MA and siBecn1 were added to inhibit autophagy. Autophagosomes are indicated by arrowheads. Representative images and statistical data on autophagosome counts are shown. **K** Western blotting was used to detect the expression level of SQSTM1-P62, MAP1LC3B, and GAPDH in NHAs treated with PBS, siNC, and H-GDEs derived from P3#GBM for 48 h; 3-MA and siBecn1 were added to the H-GDEs-treated group to inhibit autophagy. (Data reflects the mean ± SEM.*P < 0.05; **P < 0.01; ***P < 0.001; *n* = 3). GBM glioblastoma multiforme, H-GDE hypoxic glioma-derived exosome, N-GDE normoxic glioma-derived exosome, NHA normal human astrocyte, TEM transmission electron microscopy.

To examine the effects of GDEs on the function of astrocytes, GDEs from U251 and P3#GBM cells cultured under normoxic (N-GDEs) and hypoxic (H-GDEs) conditions were extracted. Transmission electron microscopy (TEM) showed that the contents derived from the supernatant were rounded objects with diameter of 30–100 nm (Fig. 1A), indicating the successful extraction of exosomes. Moreover, western blotting revealed an enriched expression of exosomal markers, including CD9 and TSG101, in the extracted vesicles, whereas calnexin was not detected (Fig. 1B). When co-cultured with NHA, exosomes labeled with PKH67 were observed in the cytoplasm of NHA cells, suggesting that exosomes could be absorbed by NHA cells (Fig. S1A). The exosome concentration was determined using nanoparticle tracking analysis with ZetaView (Particle Metrix; Fig. S1B).

Because of the hypothesis that defective autophagy triggers reactive astrocytes, therefore, the effects of GDEs on autophagy in astrocytes were investigated. The N-GDEs and H-GDEs collected from U251 and P3#GBM cells, and PBS were added to the culture media of NHAs. TEM was used to measure the formation of autophagosomes. The volume of autophagosomes significantly increased after H-GDE treatment compared with that after N-GDE treatment (Fig. 1C, D). The expression of the autophagy-related molecules, LC3B and P62, were detected in NHAs using western blotting (Fig. 1E). Both N-GDEs and H-GDEs increased autophagy in NHAs, with a greater increase in astrocytes treated with H-GDEs, indicating that GDEs directly affect autophagy in astrocytes. Similarly, LC3B within astrocytes increased upon exposure to H-GDEs sourced from U251 (Fig. S2A) and P3#GBM (Figs. 1F, S2C) based on immunofluorescent staining. The inhibition of autophagy using 3-methyladenine (3-MA) attenuated the expression of LC3B in H-GDE-treated NHAs, further confirming that H-GDEs promote autophagy in astrocytes (Figs. 1G, S2D). Additionally, siRNA-mediated knockdown of beclin1 was conducted and the efficacy of this knockdown was validated using western blotting (Fig. S2A; si-1 was used in the following experiments) as an alternative approach to inhibit autophagy. Consistent with the 3-MA treatment, the knockdown of beclin1 resulted in a similar downregulation of LC3B (Figs. 1H, S2D). Notably, both 3-MA and beclin1 silencing effectively reversed H-GDE-induced autophagosome assembly (Fig. 1I, J). Furthermore, western blotting revealed the anticipated downregulation of LC3B and concurrent upregulation of P62 following treatment with these inhibitors (Fig. 1K). Collectively, these findings highlight the ability of H-GDEs to induce autophagy in astrocytes.

### H-GDEs induce a stronger activation of NHAs than that with N-GDEs

**Fig. 2:**
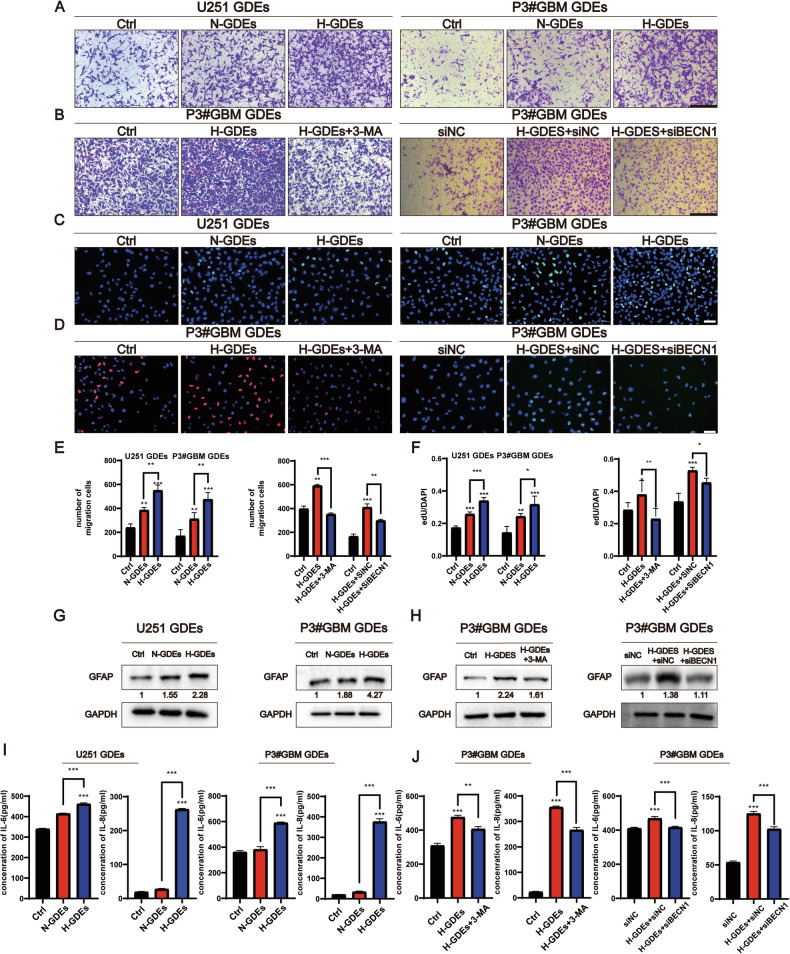
H-GDEs promoted a higher level of NHA activation than N-GDEs. **A**, **B** Migration of cells passing through the Transwell chambers of NHAs treated with PBS, N-GDEs, and H-GDEs derived from U251 and P3#GBM cells was measured using microscopy. siBecn1 and 3-MA were used to inhibit the autophagy, and representative images are shown (scale bar, 200 μm, *n* = 3). **C**, **D** An EdU assay was used to detect the proliferation of NHAs treated with PBS, N-GDEs, and H-GDEs derived from U251 and P3#GBM cells. siBecn1 and 3-MA were used to inhibit the autophagy, and representative images are shown (scale bar, 70 μm, *n* = 5). **E** Quantitative analysis of migration cell numbers. Presented here is the numerical quantification of migrated cell counts captured in (**A**, **B**), following a 48-h incubation period. **F** Measurement of EdU assay ratio. Presented here is the quantitative evaluation of the ratio of EdU-positive cells, as observed in (**C**, **D**), following a 48-h incubation period. **G**, **H** Protein collected from NHAs treated with PBS, siNC, N-GDEs, and H-GDEs derived from U251 and P3#GBM cells was used to detect the expression of GFAP and GAPDH through western blotting to detect the activation of astrocytes; siBecn1 and 3-MA were used to inhibit the autophagy. Numeric annotations below each blot represent the mean GFAP expression values from three replicate experiments, quantified by grayscale analysis. Levels are normalized to a control set at an arbitrary value of 1. **I**, **J** IL-6 and IL-8 ELISA kits were used to measure the secretion ability of NHAs treated with PBS, siNC, N-GDEs, and H-GDEs derived from U251 and P3#GBM cells; siBecn1 and 3-MA were used to inhibit autophagy. The statistical results are shown (*n* = 3). (Data reflects the mean ± SEM. *P < 0.05; **P < 0.01; ***P < 0.001). GBM glioblastoma multiforme, H-GDE hypoxic glioma-derived exosome, N-GDE normoxic glioma-derived exosome, NHA normal human astrocyte.

Given that GDEs can induce autophagy in NHA, the subsequent effects of GDEs on astrocyte function was determined. Using EdU and Transwell assays, we found that both N-GDEs and H-GDEs enhanced the proliferation and migration of NHAs, with H-GDEs exhibiting stronger effects than N-GDEs (Fig. 2A, C, E). Enzyme-linked immunosorbent assay (ELISA) showed that the increase in the proliferation and migration of NHAs was accompanied by a significant increase in the secretion of IL-6 and IL-8 in H-GDE-treated cells (Fig. 2I), indicating the transformation of NHAs into reactive astrocytes [28, 29] by H-GDEs. GFAP is a canonical marker of reactive astrocytes [30], therefore, GFAP expression was assessed after treatment with N-GDEs and H-GDEs. The findings revealed pronounced upregulation of GFAP expression in NHAs following exposure to H-GDEs (Fig. 1G), substantiating the potent pro-reactive role of H-GDEs in astrocyte activation.

GDEs promoted both autophagy and the transformation of NHAs to RAs. Next, whether autophagy played a role in mediating this transformation was investigated. Inhibition of autophagy by 3-MA or knocking down of beclin1 in the H-GDE-treated group attenuated the H-GDE-induced secretion of IL-6 and IL-8 (Fig. 2J). Whether the functional changes observed in NHAs upon GDEs exposure were mediated by autophagy was then investigated. EdU and Transwell assays also showed that the downregulation of autophagy blocked the effects of H-GDEs on the proliferation and migration of NHAs (Fig. 2B, D, F). Additionally, western blotting revealed that autophagy inhibition, achieved either through 3-MA or genetic knockdown of beclin1, led to a significant decrease in GFAP expression (Fig. 2G). Collectively, these results strongly suggest that H-GDEs can induce the transformation of NHAs into a reactive phenotype by regulating autophagy.

### H-GDEs induce autophagy in NHAs by miR-423-3p and the effect can be inhibited by 3-MA

**Fig. 3:**
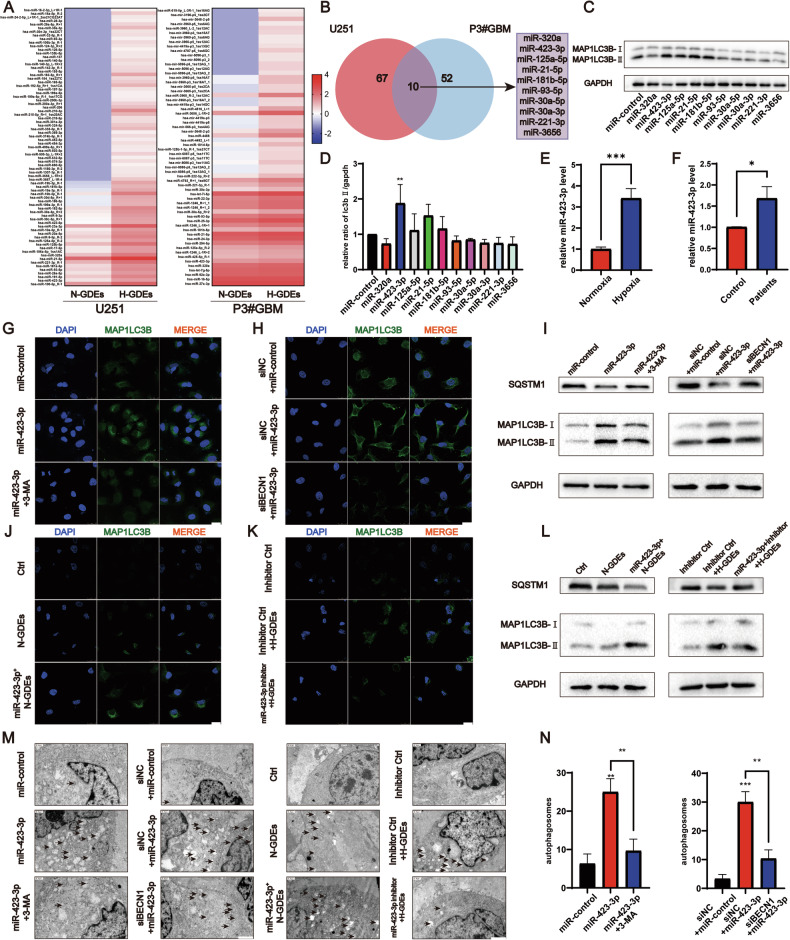
H-GDEs induced autophagy in NHAs by miR-423-3p and could be inhibited by 3-MA. **A** MiRNA array for normoxic and hypoxic exosomes derived from U251 and P3#GBM cells; the upregulated miRNAs are shown as heat map. **B** Venn diagram for the intersection of differences in exosomes in both cell lines above. **C**, **D** Western blot for candidate miRNAs inducing autophagy intersected above. Representative images of three sets of experiments and the statistical results of relative grayscale ratio of LC3B II/GAPDH are shown. **E** A qRT-PCR analysis to detect the relative expression of miR-423-3p in GDEs from P3#GBM. **F** A qRT-PCR analysis to detect the relative expression of miR-423-3p in exosomes derived from the patients with GBM and healthy patients. Statistical results of three groups are shown. **G**, **H** Immunofluorescence staining for MAP1LC3B (green) to measure the level of autophagy in NHAs transfected with miR-control and miR-423-3p; 3-MA and siBecn1 were used to inhibit autophagy in NHAs and cell nuclei were stained with DAPI (blue). Representative images of three sets of experiments are shown (scale bar, 25 μm). **I** NHAs were transfected with miR-control and miR-423-3p; 3-MA and siBecn1 were used to inhibit autophagy in NHAs. The relative expression of SQSTM1-P62, MAP1LC3B, and GAPDH in NHAs was detected using western blotting. **J**, **K** Immunofluorescence staining for MAP1LC3B (green) to detect the level of autophagy in NHAs treated with PBS, inhibitor control, N-GDEs, and H-GDEs derived from P3#GBM; miR-423-3p plasmid was transfected into P3#GBM to get miR-423-3p overexpression exosomes under normoxic conditions. miR-423-3p inhibition RNA was transfected to inhibit miR-423-3p in NHAs and cell nuclei were stained by DAPI (blue). Representative images of three sets of experiments are shown (scale bar, 25 μm). **L** NHAs were treated with PBS, inhibitor control, N-GDEs, and H-GDEs derived from P3#GBM; miR-423-3p plasmid was transfected into P3#GBM to get miR-423-3p overexpression exosomes under normoxic conditions. miR-423-3p inhibition RNA was transfected to inhibit miR-423-3p in NHAs. The relative expression of SQSTM1-P62, MAP1LC3B, and GAPDH in NHAs was detected using western blotting. **M**, **N** TEM images illustrating autophagosome formation in NHAs treated with miR-control, miR-423-3p, inhibitor control, PBS, N-GDEs, and H-GDEs derived from P3#GBM; miR-423-3p plasmid was transfected into P3#GBM to get miR-423-3p overexpression exosomes under normoxic conditions. miR-423-3p inhibition RNA was transfected to inhibit miR-423-3p in NHAs. Autophagosomes are indicated by arrowheads. Representative images and statistical data on autophagosome counts are shown. (Data reflects the mean ± SEM. *P < 0.05; **P < 0.01; ***P < 0.001; *n* = 3). GBM glioblastoma multiforme, H-GDE hypoxic glioma-derived exosome, N-GDE normoxic glioma-derived exosome, NHA normal human astrocyte, TEM transmission electron microscopy.

Exosomes can transport miRNAs from cell to cell [18]. To determine the miRNA responsible for the GDE-induced transformation of NHAs, the miRNA array was used to analyze the miRNA contents in N-GDEs and H-GDEs derived from U251 and P3#GBM cells (Fig. 3A). The differentially expressed miRNAs in N-GDEs were compared with those in H-GDEs and the top 10 miRNAs that were highly expressed in H-GDEs extracted from both cell lines were identified (Fig. 3B, *P* < 0.001, fold-change > 1). The effects of the 10 miRNAs on the regulation of autophagy in NHAs were determined. MiR-423-3p significantly elevated the expression of LC3B in NHAs, as measured semi-quantitatively (Fig. 3C, D), indicating its important role in modulating autophagy. Quantitative real-time PCR (qRT-PCR) was performed on N-GDEs and H-GDEs derived from U251 (Fig. S3A) and P3# GBM cells (Fig. 3E), which confirmed the upregulation of miR-423-3p. Additionally, the expression level was higher in the exosomes collected from the blood of patients with GBM compared with those collected from healthy patients (Fig. 3F). To further test whether miR-423-3p regulates autophagy in NHAs, 3-MA was used to inhibit autophagy in NHAs transfected with miR-423-3p mimics. qRT-PCR was performed on miR-423-3p-transfected NHAs to validate the increase in miR-423-3p expression (Fig. S3B). In addition, miRDB (http://www.mirdb.org/) and TargetScan (https://www.targetscan.org/) were used to predict the potential downstream target genes of miR-423-3p. Together with a previous study [31], both predictions showed that cytoplasmic poly(A)-binding protein-1 (*PABPC1*) and *RAP2C*, members of the RAS oncogene family, are downstream target genes of miR-423-3p. Therefore, qRT-PCR was conducted to quantify the expression of both genes in the miR-423-3p-transfected group (Fig. S3C), which further confirmed successful transfection. Western blotting and immunofluorescence images showed that miR-423-3p increased autophagy in NHAs (Figs. 3G, I, and S4A). The same trend was observed when beclin1 was knocked down in miR-423-3p-transfected cells (Figs. 3H, I and S4B), indicating the autophagic induction effect of miR-423-3p.

Given that miRNA-423-3p can induce autophagy, whether miR-423-3p mediates the effects of GDEs on autophagy was investigated. Using lentivirus-mediated expression of miR-423-3p in the P3#GBM cells, exosomes were extracted from miR-423-3p-overexpressing and negative control cells under normoxic conditions. The RNA of the derived exosomes was used to perform qRT-PCR assays, which confirmed an increase in miR-423-3p expression in the overexpression group (Fig. S3D). The extracted GDEs significantly upregulated LC3B in the NHAs when compared with that of the controls (Figs. 3J, L and S4C), and inhibitory RNA against miR-423-3p (Fig. S3E) abolished the upregulation of LC3B in the NHAs, as shown using western blotting (Fig. 3L). Immunofluorescence staining of LC3B showed similar results (Figs. 3K and S4D). Morphologically, miR-423-3p induced the formation of autophagosomes, which was inhibited by 3-MA, knockdown of beclin1, and the miRNA inhibitor (Figs. 3M, N and S4E, F). Collectively, miR-423-3p plays an important role in inducing autophagy in NHAs.

### MiR-423-3p-induced autophagy in H-GDEs promotes the activation of astrocytes

**Fig. 4:**
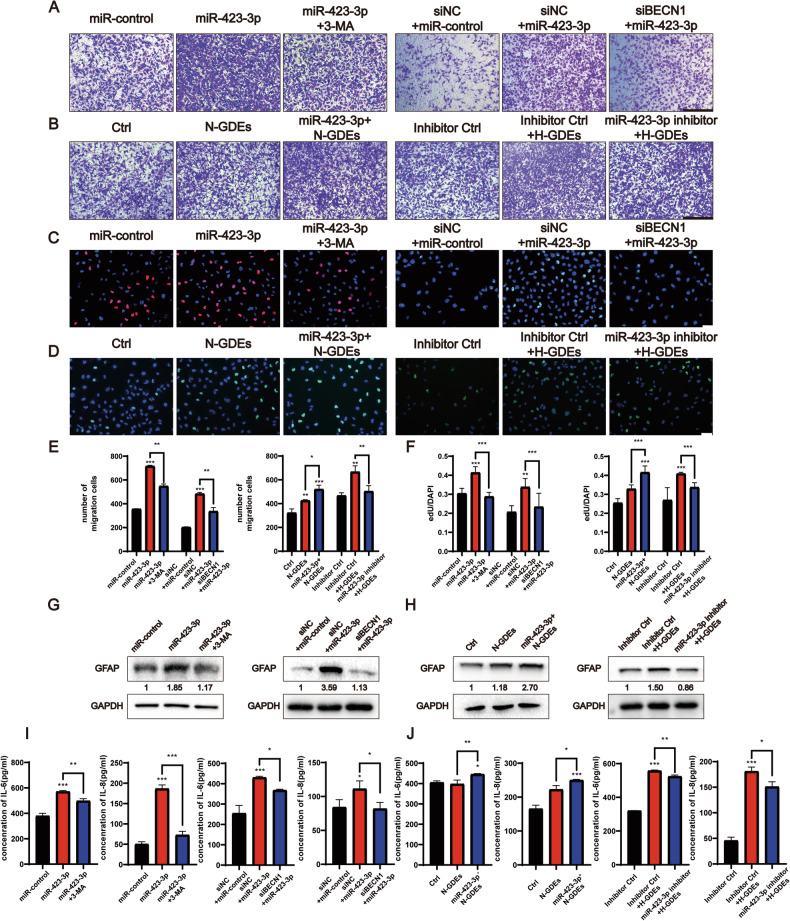
MiR-423-3p-induced autophagy in H-GDEs promotes the activation of astrocytes. **A**, **B** Migration of cells passing through the Transwell chambers of NHAs treated with miR-control, miR-423-3p, inhibitor control, PBS, N-GDEs, and H-GDEs; 3-MA and siBeclin1 were used to inhibit autophagy. The miR-423-3p plasmid was transfected into P3#GBM to obtain miR-423-3p-overexpressing exosomes under normoxic conditions. MiR-423-3p inhibitory RNA was transfected to inhibit miR-423-3p in NHAs, and representative images are shown (scale bar, 200 μm, *n* = 3). **C**, **D** EdU assay was used to detect the proliferation ability of NHAs clarified in (**A**, **B**) and representative images are shown (scale bar, 70 μm, *n* = 3). **E** Quantitative analysis of migration cell numbers. The numerical quantification of migrated cell counts captured in Figures A and B, following a 48-h incubation period. **F** Measurement of EdU assay ratio. Quantitative evaluation of the ratio of EdU-positive cells, as observed in (**C**, **D**), following a 48-h incubation period. **G**, **H** Protein collected from NHAs clarified in (**A**, **B**) was used to detect the expression of GFAP and GAPDH through western blotting to detect the activation of astrocytes. Numerical annotations below each blot denote the average GFAP expression values from three replicate experiments, quantified by grayscale analysis. Expression levels are normalized to a control set at 1. **I**, **J** Cell supernatant from NHAs clarified in (**A**, **B**) was collected to detect the secretion of cytokines through IL-6 and IL-8 ELISA kits. The statistics were measured by a microplate reader (*n* = 3). (Data reflects the mean ± SEM. *P < 0.05; **P < 0.01; ***P < 0.001). GBM glioblastoma multiforme, H-GDE hypoxic glioma-derived exosome, N-GDE normoxic glioma-derived exosome, NHA normal human astrocyte.

MiR-423-3p induce autophagy in NHAs, therefore, whether it promoted astrocyte transformation was explored. NHAs were transfected with miR-423-3p mimics and treated with or without autophagy inhibition using 3-MA or beclin1 knockdown RNA. Transwell and EdU assays showed that miR-423-3p significantly increased the proliferation and migration of NHAs, which was attenuated by autophagy inhibition (Fig. 4A, C, E, F). Using ELISA and western blotting, we confirmed that miR-423-3p increased the secretion of IL-6 and IL-8 from astrocytes (Fig. 4I), and upregulated GFAP expression (Fig. 4G). These results indicate that miRNA-423-3p promotes the transition of NHA to RAs. To further examine whether miR-423-3p mediates the effects of GDEs on transformation of NHAs, GDEs were extracted from the P3#GBM cells overexpressing miR-423-3p under normoxic conditions to treat the NHAs. H-GDEs with an miRNA that inhibits miR-423-3p were added to the other group. MiR-423-3p significantly enhanced the transformation, whereas inhibitory RNA rescued the H-GDE-induced transformation of NHAs (Fig. 4B, D–F, H, J). Together, these results demonstrate that miR-423-3p in H-GDEs promotes the tumor-promoting transformation of NHAs through autophagy.

### RAs induced by miR-423-3p in H-GDEs promote the progression of glioma in vivo

**Fig. 5:**
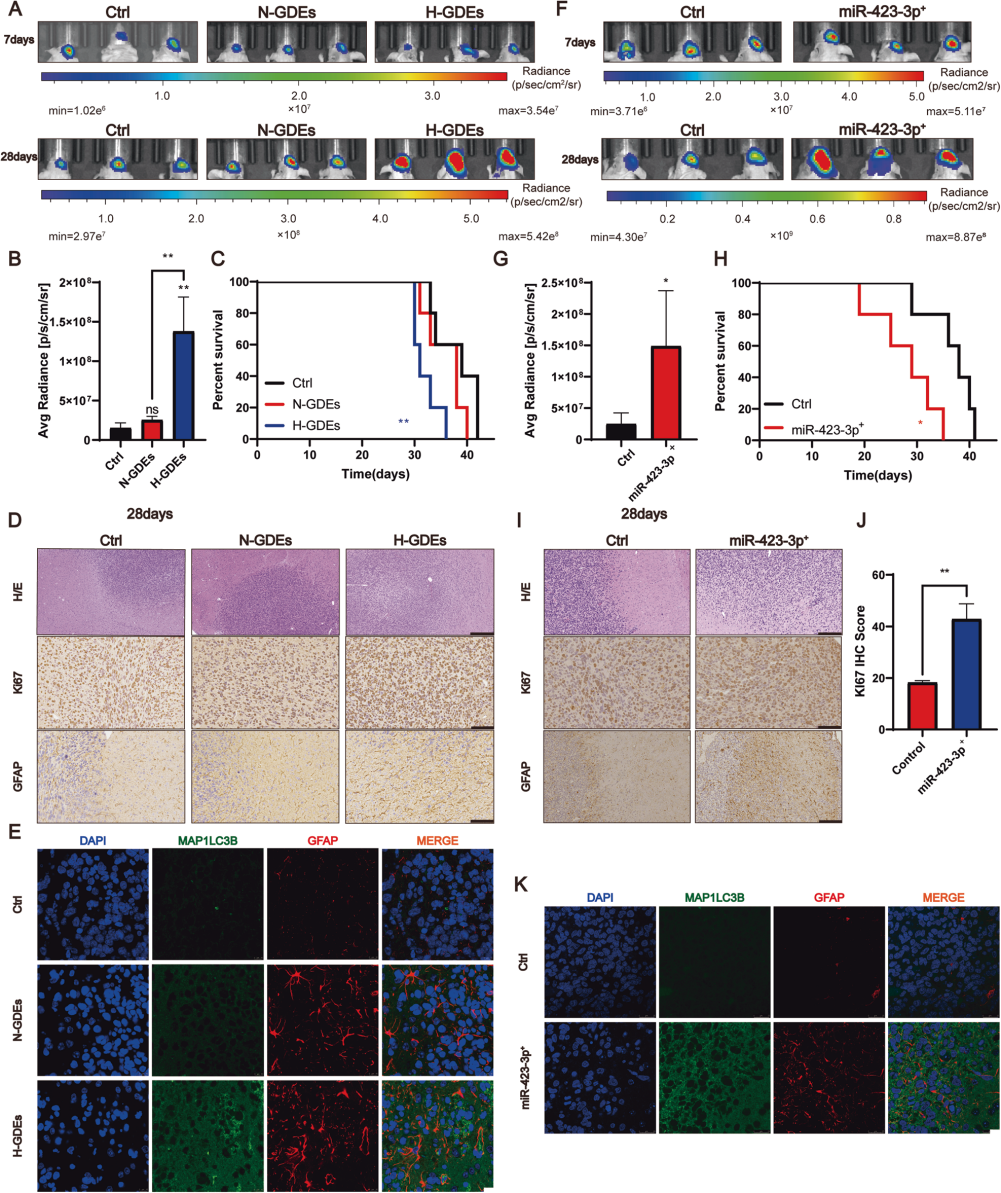
RAs induced by miR-423-3p in H-GDEs promoted the progression of glioma in vivo. **A**, **B** Bioluminescence imaging of tumor-bearing mice treated with P3#GBM together with PBS-NHA, N-GDEs-NHA, and H-GDEs-NHA. Representative images and statistical analysis of day 28 are shown. **C** Survival analysis for orthotopic xenografts bearing P3#GBM together with PBS-NHA, N-GDEs-NHA, and H-GDEs-NHA (five mice per group). **D** H&E staining (scale bar, 300 μm) and IHC staining (scale bar, 100 μm) for Ki67 and GFAP from xenograft mice treated with P3#GBM, PBS-NHA, N-GDEs-NHA, and H-GDEs-NHA on the day of euthanasia. Representative images of three sets of experiments are shown. **E** Co-immunofluorescence staining for LC3B (green) and GFAP (red) in NHAs in orthotopic xenografts treated with PBS, N-GDEs, and H-GDEs derived from P3#GBM after euthanasia. Cell nuclei were stained with DAPI (blue). Representative images of three sets of experiments are shown (scale bar, 10 μm). **F**, **G** Bioluminescence imaging of tumor-bearing mice treated with P3#GBM together with lenti-control-NHA and lenti-ov-miR-423-3p-NHA. Representative images and statistical analysis of day 28. **H** Survival analysis for orthotopic xenografts bearing P3#GBM together with lenti-control-NHA and lenti-ov-miR-423-3p-NHA (five mice per group). **I**, **J** H&E staining (scale bar, 300 μm) and IHC staining (scale bar, 100 μm) for Ki67 and GFAP from xenograft mice co-planted with lenti-control-NHA and lenti-ov-miR-423-3p-NHA. Representative images of three sets of experiments and statistical analysis for IHC score of Ki67 are shown. **K** Co-immunofluorescence staining for LC3B (green) and GFAP (red) in lenti-control-NHAs and lenti-ov-miR-423-3p-NHAs. Cell nuclei were stained with DAPI (blue). Representative images of three sets of experiments are shown (scale bar, 10 μm). (Data reflects the mean ± SEM. *P < 0.05; **P < 0.01; ***P < 0.001; *n* = 3). GBM glioblastoma multiforme, H-GDE hypoxic glioma-derived exosome, N-GDE normoxic glioma-derived exosome, NHA normal human astrocyte, RA reactive astrocytes.

Given that glioma cells can induce the transformation of NHAs to RAs through exosomal-based mechanisms, whether the transformation of astrocytes by GDEs or miR-423-3p can promote the progression of glioma was investigated. P3#GBM cells and astrocytes pretreated with PBS, N-GDEs, and H-GDEs were co-implanted into the brains of nude mice and treated the mice with PBS, N-GDEs, and H-GDEs through the tail vein every 3 days. The tumor volume measured by bioluminescence imaging at one and 4 weeks post implantation showed that the size of the tumors in mice treated with H-GDE was significantly larger than that in mice in the other two groups (Figs. 5A, B and S5A). Subsequently, mice treated with H-GDEs exhibited a shorter survival time than mice in the other groups (Fig. 5C). H&E and IHC staining of Ki67 showed that the tumors in the H-GDE-treated group had few clear borders and high Ki67 expression. The astrocyte marker, GFAP, was also stained; NHAs treated with H-GDEs exhibited a high expression of heteromorphic GFAP, with a clear increase in the number of branches (Figs. 5D, S5B). Moreover, co-immunostaining of LC3B and GFAP showed that H-GDE treatment significantly increased the expression of LC3B and abnormal GFAP in NHAs (Fig. 5E), suggesting that H-GDEs could promote autophagy and transformation in NHAs in vivo.

After confirming the role of H-GDEs in facilitating glioma progression in vivo, the effects of miR-423-3p on tumor progression was examined. NHAs were transferred with lenti-miR-control or lenti-ov-miR-423-3p virus, and co-implanted into nude mice alongside P3#GBM cells to generate orthotopic xenografts. Bioluminescence imaging revealed that by the 4th week, miR-423-3p increased the tumor size in nude mice (Figs. 5F, G and S5C). Moreover, the survival rate of the implanted mice was lower than that of the control mice (Fig. 5H). Consistently, H&E staining showed strong tumor infiltration in the miR-overexpressing group (Fig. 5I). Additionally, IHC staining for Ki67 and GFAP showed that the activation of NHAs induced by miR-423-3p promoted the proliferation rate of gliomas and the expression of heteromorphic GFAP in NHAs (Fig. 5I, J). Co-immunostaining further indicated that lenti-miR-423-3p-expressing mice exhibited higher levels of LC3B and heteromorphic GFAP compared with those of control mice (Fig. 5K). Collectively, these results suggest that miR-423-3p derived from H-GDEs can promote the transformation of NHAs in the TME into RAs and subsequently facilitate the progression of glioma in vivo.

### LAMP3 is the downstream target gene of miR-423-3p and participates in autophagy in NHAs

**Fig. 6:**
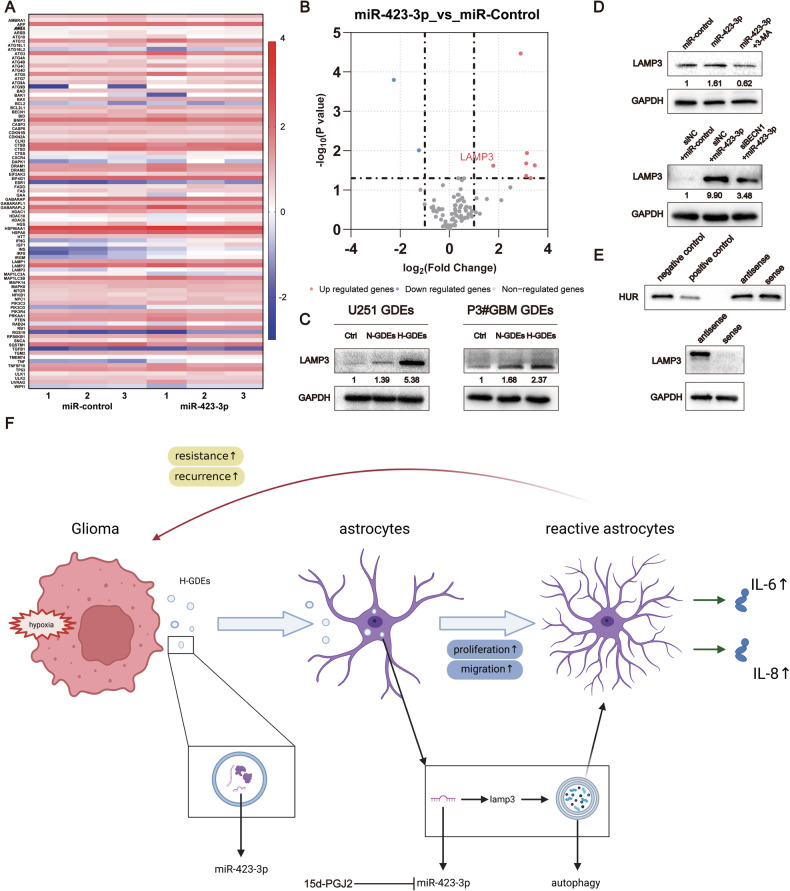
LAMP3 is a downstream target gene of miR-423-3p that participates in the autophagy of NHAs. **A**, **B** NHAs were transfected with negative control mimics and miR-423-3p mimics. Utilizing a qPCR array, the heat map and volcano map showcased differential gene expression patterns. **C**, **D** The relative expression of Lamp3 was detected using western blotting in NHAs treated with PBS, U251-derived N-GDEs, H-GDEs, P3#GBM-derived N-GDEs, H-GDEs, control mimics, siNC, and miR-423-3p mimics; 3-MA and siBeclin1 were added to inhibit autophagy respectively. Below each blot, numbers represent the mean LAMP3 expression values from three independent experiments, quantified using grayscale analysis. Expression levels are normalized to a control set at 1. **E** A pulldown kit was used for NHAs to validate the connection between miR-423-3p and Lamp3; expression of human antigen R (HUR) was measured in negative and positive proteins (derived from the pulldown kit), and the NHA protein was added with biotin-marked sense and antisense RNA to confirm the successful pulldown of proteins. The expression of LAMP3 and GAPDH was detected in NHA protein treated with biotin-marked sense and antisense RNA. **F** A schematic diagram was created using biorender.com. GBM glioblastoma multiforme, H-GDE hypoxic glioma-derived exosome, N-GDE normoxic glioma-derived exosome, NHA normal human astrocyte.

To detect the downstream genes of miR-423-3p involved in its effects on autophagy, qPCR array was used to probe the autophagy-related target genes in NHAs transfected with the control and miR-423-3p mimics (Fig. 6A, B). According to previous studies [32–35], lamp3 may be a downstream target of miR-423-3p, and its function is closely involved in autophagy. The expression of LAMP3 in NHAs treated with PBS, N-GDEs, and H-GDEs derived from U251 and P3#GBM cells were compared using western blotting. H-GDEs significantly increased the expression of LAMP3 in NHAs (Fig. 6C). Moreover, in miR-423-3p-transfected NHAs, LAMP3 expression increased, and this trend was reversed when autophagy was inhibited (Fig. 6D). These experiments indicated that LAMP3 may be involved in the effects of miR-423-3p on the induction of autophagy. A pull-down assay showing a direct interaction between miR-423-3p and LAMP3 further supported this speculation (Fig. 6E).

### The 15d-PGJ2 can inhibit the activity of miR-423-3p and suppress the activation of NHAs

**Fig. 7:**
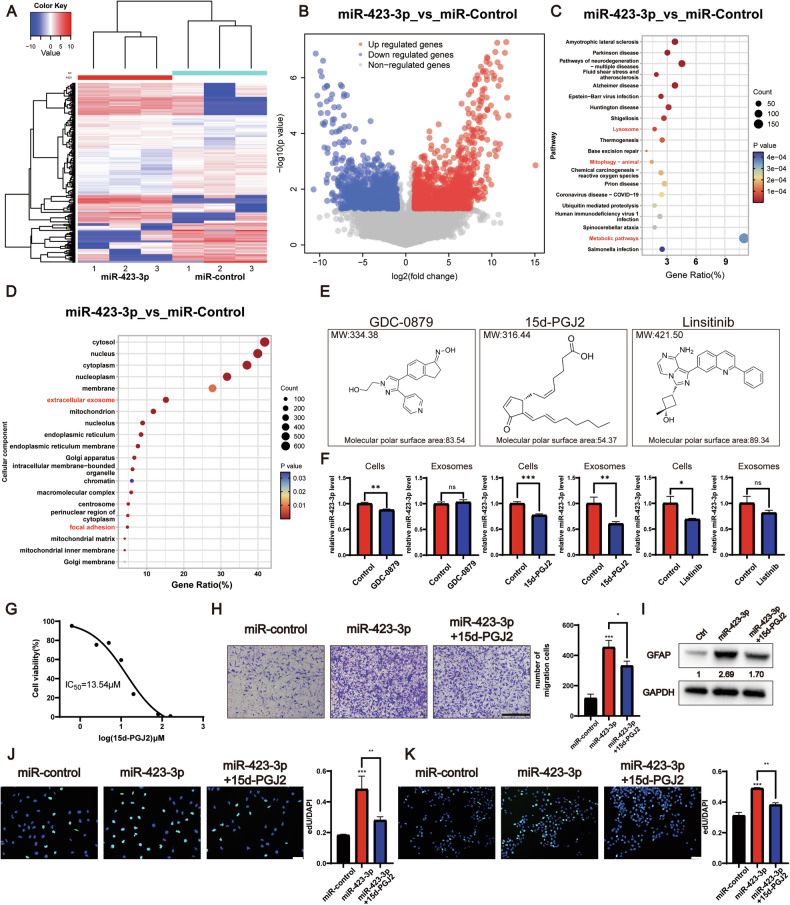
15d-PGJ2 can inhibit the activity of miR-423-3p and suppress the activation of NHAs. **A** Heatmap depicting the cluster analysis of differentially expressed genes in NHAs treated with miR-control versus miR-423-3p mimics, illustrating distinct gene expression patterns. **B** Volcano plot showcasing differential gene expression in NHAs following treatment with miR-control and miR-423-3p mimics (|log2FC| ≥ 1 and *P* < 0.05). **C** Dot plot representing KEGG pathway enrichment analysis of transcriptome sequencing data from NHAs treated with miR-control and miR-423-3p mimics, indicating enriched pathways. **D** Dot plot summarizing the cellular component aspect of GO enrichment analysis derived from transcriptome sequencing in NHAs treated with miR-control and miR-423-3p mimics. **E** Schematic diagrams of the three top-ranked inhibitors of miR-423-3p, commercially available and predicted to cross the blood-brain barrier. **F** qRT-PCR analysis revealing the relative expression levels of miR-423-3p in P3#GBM cells and exosomes following treatment with 5 μM GDC-0879, 15d-PGJ2 and linsitinib, respectively. **G** Survival curves for NHAs exposed to increasing concentrations 15d-PGJ2 (0.6–160 μM) over 48 h to determine the IC50 values. **H** Representative images and statistical analysis showing the migration capacity of NHAs across Transwell chambers treated with miR-control, miR-423-3p, and 15d-PGJ2 (scale bar, 200 μm). **I** Western blotting of protein extracts from NHAs treated with miR-Control, miR-423-3p, and 15d-PGJ2, demonstrating the expression of GFAP and GAPDH. Relative GFAP levels of three replications are quantified via grayscale analysis with control set to 1. **J** EdU assay demonstrates NHAs proliferative response to miR-Control, miR-423-3p, and 15d-PGJ2 treatments. Representative images and statistical analysis are shown (scale bar, 70 μm) **K** EdU assay was used to detect the proliferation ability of P3#GBM co-cultured with NHAs pretreated with miR-control, miR-423-3p, and 15d-PGJ2. Representative images and statistical analysis are shown (scale bar, 70 μm) (Data reflects the mean ± SEM. ns *P* ≥ 0.05*P < 0.05; **P < 0.01; ***P < 0.001; *n* = 3). GBM glioblastoma multiforme, NHA normal human astrocyte.

To identify potential drugs that can inhibit miR-423-3p activity, transcriptome sequencing of NHAs treated with miR-control or miR-423-3p was conducted, which allowed us to identify differentially expressed genes (Fig. 7A, B). KEGG enrichment analysis revealed that the differentially expressed genes were associated with lysosomal function, mitophagy, and metabolic pathways, all of which are associated with autophagy (Fig. 7C). Additionally, GO enrichment analysis highlighted exosomes and focal adhesions, contributing to the subsequent formation of the glioma microenvironment (Figs. 7D, S6A). The CMap database was used to predict potential drugs based on the differentially expressed genes. The top three drugs predicted to pass through the blood-brain barrier (BBB) were GDC-0879, 15d-PGJ2, and linsitinib (Fig. 7E). At a concentration of 5 μM, 15d-PGJ2 exhibited potent inhibitory effects on both glioma cells and exosomes, distinguishing its efficacy compared with that of GDC-0879 and linsitinib (Fig. 7F). Together with the IC50 tested using CCK-8 (Figs. 7G, S7A), the molecular topological polar surface area, and molecular weight of these candidates, 15d-PGJ2 was chosen to conduct the following experiments.

15d-PGJ2 is a kind of selective peroxisome proliferator-activated receptor gamma (PPARγ) and a covalent PPARδ agonist. It could inhibit the proliferation and expansion of glioma [36]. In NHAs, 15d-PGJ2 treatment led to a decrease in migration rate (Fig. 7H), level of GFAP (Fig. 7I), and proliferation rate (Fig. 7J) induced by miR-423-3p. Additionally, the activation induced by H-GDEs was also inhibited by 15d-PGJ2 in the NHAs (Fig. S7B–D). Subsequently, the proliferation rate of P3#GBM cells was observed to decrease when co-cultured with NHAs pretreated with 15d-PGJ2 (Fig. 7K), highlighting the potential anti-proliferative effects of this compound on GBM cell growth within a complex cellular microenvironment. Thus, 15d-PGJ2 was predicted to be a promising inhibitor for decreasing the activation of NHAs and formation of the TME.

## Discussion

Although various treatment methods have been used clinically, the prognosis of patients with GBM is poor owing to high treatment resistance and recurrence rates [37, 38]. An important factor in drug resistance and recurrence is the TME, which is formed by various cells influenced by tumors, such as tumor-associated macrophages [39], neurons, astrocytes, and microglia [40]. Astrocytes, one of the major sources of gliomas according to the International Classification of Diseases–Oncology, version 3 and the World Health Organization grade [4, 41, 42], play an essential role in remodeling the TME. Indeed, studies has reported that astrocytes directly or indirectly regulate the progression and drug resistance in gliomas [43, 44]. However, whether and how the crosstalk between glioma cells and astrocytes contributes to malignant changes in the TME remains poorly understood. Exosomes act as mediators of intercellular communication in the TME and promote tumor cell progression in various cancers [45, 46]. Furthermore, hypoxia, which results from the disparate growth rates of tumor cells and blood vessels, is a key characteristic of gliomas. It promotes tumor progression and, more importantly, influences the formation and heterogeneity of tumor-derived exosomes, subsequently contributing to the development of an immunosuppressive TME [47]. Based on these observations, we compared N-GDE and H-GDE effects on the induction of RAs. H-GDEs induced a much stronger reactive phenotype in astrocytes, accompanied by increased proliferation, migration, cytokine secretion, and GFAP expression. Furthermore, when H-GDE-treated astrocytes were co-implanted with glioma cells in vivo, a significantly higher proliferation rate of glioma than that of the N-GDE group. Consistent with previous studies, hypoxia-derived exosomes function as mediators of intracellular communication in the TME. For example, Qiu et al. reported that H-GDEs led to the increased differentiation and activation of myeloid-derived suppressor cells by increasing the content of miR-1246 in exosomes [48]. In gliomas, H-GDEs can induce the M2-polarization of macrophages by directing IL-6 into macrophages [18]. Exosomes have also been reported to induce the activation of astrocytes. Exosomes containing miR-21 secreted by neurons contributed to the overactivation of astrocytes in a mouse model of Alzheimer’s disease [49]. Therefore, the exosomes mediate the intracellular communication between glioma and astrocytes and transform the detrimental effects of hypoxic conditions on TME remodeling. Exosomes participate in message exchange between tumor cells and other cell types in the TME by delivering tumor-derived contents to recipient cells [50]. Among the genetic contents delivered by exosomes, miRNAs have specific and important effects on various biological activities of cells, such as proliferation, DNA repair, and feedback regulation [51, 52]. Therefore, we used miRNA arrays to identify potential miRNAs that mediate the effects of GDEs on RA formation. After comparing the differentially regulated miRNAs between H-GDEs and N-GDEs, miR-423-3p was significantly upregulated in H-GDEs. Next, the effect of miR-423-3p on the induction of RAs was examined. Similar to the findings of H-GDEs, miR-423-3p-transfected NHAs exhibited a significant increase in the proliferation, migration, and secretion of proinflammatory cytokines, accompanied by elevated levels and atypia of GFAP. Compared to the effect of N-GDEs from control cells, N-GDEs with stabilized expression of miR-423-3p resulted in a much more reactive phenotype of RAs, imitating the effect of H-GDEs. In contrast, when miR-423-3p was inhibited in NHAs, the effect of H-GDEs was reduced. Therefore, miR-423-3p is a key component that mediates the effects of GDEs on RAs induction. The in vitro xenograft model further confirmed these findings. The volume of glioma cells co-implanted with astrocytes transfected with miR-423-3p was significantly higher than that of mice in the control group, leading to a greater reduction in the survival time of mice. Consistently, miR-423-3p has been reported to promote the progression of gliomas [18] as well as various other tumors, including gastric cancer, hepatocellular carcinoma, and colorectal cancer [53–55], indicating its functional role in promoting malignancy. These results strongly suggest the key role miR-423-3p plays in astrocyte activation.

In addition, the role of miRNAs in neurodegenerative disorders has gained considerable attention [56, 57]. For instance, octadecaneuropeptide alleviates the formation of reactive astrocytes induced by 6-hydroxydopamine by downregulating the overexpression of miR-21 in models of Parkinson’s disease models [58]. Furthermore, downregulation of the miR-106b and miR-101 in patients with Alzheimer’s disease results in the expression of amyloid precursor protein and the generation of Aβ, resulting in the activation of surface receptors on astrocytes and aggravation of Alzheimer’s disease [59]. As for miR-423-3p, Zameer et al. elucidated that miR-423-3p is markedly upregulated and associated with several high-risk genes linked to multiple Parkinson’s disease genes [60]. In amyotrophic lateral sclerosis, alterations in the levels of miR-423-3p serve as critical biomarkers for the early stages of the disease [61]. These results strongly suggest that miR-423-3p plays an important role in the occurrence and development of central nervous system diseases.

Autophagy is an important regulatory process that maintains cellular homeostasis. However, in tumor cells, their functions may vary under different conditions. In the early stages of tumor formation, autophagy can help eliminate tumor cells and inhibit their progression; however, when autophagy is dysregulated, it promotes the survival and growth of tumor cells [62, 63]. Autophagy is involved in the crosstalk between tumors and other cell types in the TME [64], suggesting that autophagy can also contribute to the formation of a malignant TME. The autophagy levels in NHAs treated with H-GDEs and N-GDEs were detected. Morphologically, autophagosome formation in astrocytes of the H-GDE-treated group increased. The expression level of LC3B in the H-GDE group was higher than that in the N-GDE group and the expression of P62 was the opposite to that of LC3B, indicating an increase in autophagy. Next, whether miR-423-3p is a direct messenger that induces autophagy in NHAs was examined. MiR-423-3p induced autophagy in NHAs, including the formation of autophagosomes, an increase in LC3B, and a decrease in P62. Similar to this study, Zhang et al. reported that the upregulation of miR-2188-5p increases the autophagic flux of tracheal cells in an ammonia environment [65]. In another study, miR-122 was shown to induce protective autophagy in hepatocytes under arsenic stress [66]. These studies have confirmed the role of miRNAs in the regulation of autophagy. However, whether autophagy promotes astrocyte activation remained unclear. Using the autophagy inhibitor 3-MA or knocking of beclin1, NHAs treated with H-GDEs or miR-423-3p mimicked the phenotype observed with autophagy inhibition. The typical properties of RAs were also attenuated, suggesting that H-GDEs or miR-423-3p induced RAs through pathological changes by inducing autophagy. However, most published studies suggest that the activation of astrocytes is induced by the inhibition of autophagy, which is inconsistent with to the results of this study. However, as clarified above, autophagy may have different effects on tumors under different periods and circumstances. Furthermore, astrocytes are considered one of the origins of gliomas. A reactive procedure is required when astrocytes are malignantly induced in glioma cells. Therefore, in some situations, autophagy in NHAs may be increased to induce RAs, leading to tumor progression. In addition, a previous study reported that in the TME and distal tissue, tumor cells can induce non-cell-autonomous autophagy of normal cells to promote tumor progression in the early stages [67], which is consistent with this study. Moreover, most studies focusing on the formation of RAs by autophagic inhibition are non-oncological. Hence, the effects of autophagy on the TME and astrocytes may differ.

MiRNAs are generally believed to regulate gene expression by inhibiting the expression of target genes; however, sometimes, miRNAs can also promote the expression of downstream genes. This phenomenon is called “miRNA mediated transcriptional activation” or “miRNA enhancement effect.” In some cases, certain mRNAs act as a “sponge” to absorb a specific miRNA, thereby indirectly increasing the expression of other mRNAs that share the same miRNA response elements [68]. Li et al. demonstrated that miRNAs alter the chromatin state by interacting with chromatin-modifying factors, thereby promoting gene expression [69]. Furthermore, miRNAs can directly bind to transcription start sites to promote their expression [70]. According to this study, miRNAs may interact with proteins to maintain their stability. However, further studies are required to elucidate the detailed mechanisms underlying these interactions.

The formidable presence of the BBB poses a substantial impediment to the direct administration of drugs for the treatment of gliomas, necessitating the identification of agents that can effectively modulate the TME and traverse the BBB. To address this, the CMap database was used to screen for readily available compounds capable of influencing the activity of miR-423-3p. Based on the concentration of miR-423-3p and IC50 values, 15d-PGJ_2_, a PPARγ agonist derived from cyclopentenone prostaglandins, was selected as a candidate drug for further research. It inhibits tumor progression by affecting both tumor cells and the TME [71]. Specifically, it suppresses CD133+ brain tumor stem cells by downregulating the EGF/bFGF and Tyk2–Stat3 pathways [36]. Our analysis revealed that 15d-PGJ_2_ potently inhibits the activation of NHAs induced by H-GDEs or miR-423-3p. In addition, 15d-PGJ2 counteracted the enhanced proliferation of glioma cells following exposure to reactive astrocytes. Consistent with this study, previous studies have shown that primary and cyclopentenone prostaglandins, including 15d-PGJ_2_, downregulate astrocyte proliferation [72]. Moreover, 15d-PGJ_2_ inhibits IL-6 expression in pituitary cells [73], aligning with this study. These findings suggest that 15d-PGJ_2_ is a promising therapeutic agent for inhibiting glioma progression, particularly by modulating reactive astrocytes and the TME.

In summary, this study identified a positive feedback mechanism through which gliomas trigger astrocyte activation, which in turn promotes glioma malignancy. Furthermore, gliomas promoted the transformation of NHAs into RAs through the secretion of miR-423-3p-containing exosomes, which subsequently induced astrocytic reactivity through autophagy. This effect was inhibited by 15d-PGJ2. This study highlights the importance of the TME in brain tumor research, shifting the focus from only the tumor. Astrocytes and other cells in the TME may be a completely new research perspective, not only in glioma research but also in other brain research, such as neurodegenerative disorders. Additionally, this study provides new biological insights into the diagnosis and treatment of gliomas. Changes in the levels of exosomes and miR-423-3p may help in the technological improvement of liquid biopsies. The activation of reactive astrocytes can aid in the diagnosis of glioma. The inhibition of miR-423-3p and autophagy may disrupt the formation of the TME and ultimately impair glioma progression. The use of 15d-PGJ2 may help treat gliomas from a new perspective.

### Statistical analysis

All data were compared using one-way ANOVA, Student’s *t*-test et al. to detect differences using GraphPad Prism 8. Data from the experiment are shown as means ± SD. All tests were considered statistically significant at *P* < 0.05.

## Supplementary information

Supplymental Material

Supplementary Figure 1

Supplementary Figure 2

Supplementary Figure 3

Supplementary Figure 4

Supplementary Figure 5

Supplementary Figure 6

Supplementary Figure 7

original western blot

reproducibility checklist

## Data Availability

The datasets used and analyzed in the current study are available from the corresponding author upon reasonable request.
