# Supplementary material for: Inhibition of hypoxic exosomal miR-423-3p decreases glioma progression by restricting autophagy in astrocytes
Source: Cell Death Dis. 2025 Apr 8;16(1):265. doi: 10.1038/s41419-025-07576-2 (PMC11978802; doi:10.1038/s41419-025-07576-2)
Supplement: Supplementary file 1 — Supplymental Material [file 41419_2025_7576_MOESM1_ESM.docx]

**Supplemental methods**

**Electron microscopy**

The cells and exosomes were fixed with 2.5% glutaraldehyde for 2 h and washed with PBS 3 times for 15 min each time. Then, the samples were fixed with 1% osmic acid for 2 h and washed with PBS in the same procedure described above. Fixed samples were dehydrated with 50% ethanol for 15 min, 70% ethanol for 15 min, 90% ethanol for 15 min, 90% ethanol mixed with 90% acetone (1:1) for 15 min, and 90% acetone for 15 min at 4°C and fixed with 100% acetone for 15 min 3 times at room temperature. The dehydrated samples were embedded with 100% acetone and embedding fluid and solidified in a baking oven. The embedded samples were sliced and stained with 3% uranyl acetate and lead citrate. Stained samples were observed by a JEM-1200X system (JEOL Ltd., Akishima, Tokyo, Japan).

**Western blot analysis**

NHAs and exosomes were collected and treated with RIPA lysis buffer (Thermo Fisher Scientific, Waltham, MA, USA) with 2% proteinase inhibitor and 2% phosphatase inhibitor (Beyotime, Shanghai, China). The concentration of extracted protein was detected by a BCA Protein Assay kit (Beyotime). Extracted proteins were seperated with 12.5% SDS‒PAGE before transferred onto polyvinylidene difluoride (PVDF) membranes and incubated with primary antibodies against LC3B, CD9, calnexin (Cell Signaling Technology, Danvers, MA, USA), GFAP, P62, LAMP3, Beclin1, GAPDH (Proteintech, Chicago, IL, USA), and Tsg101 (Abcam, Cambridge, UK) overnight at 4°C. The membranes were incubated with the secondary antibody for 1 h at room temperature according to the species of primary antibodies. The results were visualized by chemiluminescence according to the manufacturer’s protocol.

**Pulldown assays**

The Pierce Magnetic RNA‒Protein Pull-Down Kit (Thermo Fisher Scientific, Waltham, MA, USA) was used to perform the pulldown experiment under the manufacturer’s guidance. The biotin-labeled miR-423-3p sense and antisense sequences were synthesized by GenePharma (Shanghai, China). Their sequences were as follows: sense: 5'-AGCUCGGUCUGAGGCCCCUCAGU-3', antisense: 5'-ACUAGGGGCCUCAGACCGAGCU-3'. The results were detected by western blotting.

**Immunofluorescence**

Tissues and NHA samples were fixed with 4% paraformaldehyde at room temperature for 10 min and treated with 0.3% Triton X-100 for 7 min. The treated samples were blocked with 10% goat serum for 1 h and reacted with primary antibodies against LC3B and GFAP at 4°C overnight. The primary antibodies were detected with goat anti-mouse IgG H&L (Alexa Fluor® 488) and goat anti-rabbit IgG H&L (Alexa Fluor® 647) (Abcam, Cambridge, UK). DAPI was used to detect the nuclei. The fluorescence difference was detected by confocal microscopy.

The NHAs samples used for PKH67 staining was processed by Cell membrane staining kit (PKH67) (Beijing Bio-Lab Co., Ltd. China) under the guidance of manufacturer’s protocol.

**Cytokine assay**

The cell supernatants of NHAs were collected at 48h of treatments. The secretion of IL-6 and IL-8 was detected by the AuthentiKine™ Human IL-6 ELISA Kit and Human IL-8 ELISA Kit (Proteintech, Chicago, IL, USA), respectively, according to the manufacturer’s instructions.

**RNA extraction and qRT‒PCR**

The total RNA of NHAs, P3#GBM and exosomes was extracted using the RN001 RNA- Quick Purification Kit (ES Science, Shanghai, China) and then transcribed into cDNA by High-Capacity cDNA Reverse Transcription Kits (Applied Biosystems, Foster City, CA, USA) according to the manufacturer’s protocols. Real-time PCR was performed by the quantitative PCR System Mx-3000P (Stratagene, La Jolla, CA, USA) according to the manufacturer’s protocols. The PCR sequences of primers used in the study are listed as follows: GAPDH: forward, 5'-GCACCGTCAAGGCTGAGAAC-3' and reverse, 5'-TGGTGAAGACGCCAGTGGA-3'; PABPC1: forward, 5'-GAGCTGTTCCCAACCCTGTA-3' and reverse, 5'-AGCA CCGGGCATATTTTGGA-3'; RAP2C: forward, 5'-TCCTTTCATGGAGACATCGGC-3′ and reverse, 5'-TGATCTTGCTTCTCCGGCA-3'; miR-423-3p: forward, 5'-ATAAGCTCGGTCTGAGGCCC-3' and reverse, 5'-TATCCTTGTTCACGACTCCTTCAC-3'; U6: forward: 5'-CAGCACATATACTAAAATTGGAACG-3' and reverse, 5'-ACGAATTTGCGTGTCATCC-3'.

**Viral transfection and autophagic inhibition**

The miR-423-3p overexpression and control lentiviruses were synthesized by Vigene Bioscience (Suzhou, China), and 3-MA (0.5 mM, MedChemExpress, Monmouth Junction, NJ, USA) and beclin1 (introduced in RNA transfection) were used to inhibit autophagy.

**RNA transfection**

MiRNA mimics, miRNA inhibitor, siRNA and negative controls used to transfect NHAs and P3#GBM were synthesized by GenePharma (Shanghai, China). RNAs were transfected by Lipofectamine 2000 (Thermo Fisher Scientific, Waltham, MA, USA) for 48 h when the cell density reached 70-80%. The RNA sequences were listed as follows: miR-320a mimics: sense: 5'-AAAAGCUGGGUUGAGAGGGCGA-3', antisense: 5'-GCCCUCUCAACCCAGCUUUUUU-3'; miR-30a-5p mimics: sense: 5'-UGUAAACAUCCUCGACUGGAAG-3', antisense: 5'-CUUCCAGUCGAGGAUGUUUACA-3'; miR-221-3p mimics: sense: 5'-AGCUACAUUGUCUGCUGGGUUC-3', antisense: 5'-GAAACCCAGCAGACAAUGUAGCU-3'; miR-181b-5p mimics: sense: 5'-AACAUUCAUUGCUGUCGGUGGGU-3', antisense: 5'-CCACCGACAGCAAUGAAUGUUUU-3'; miR-30a-3p mimics: sense: 5'-CUUUCAGUCGGAUGUUUGCAGC-3', antisense: 5'-UGCAAACAUCCGACUGAAAGUU-3'; miR-423-3p mimics: sense: 5'-AGCUCGGUCUGAGGCCCCUCAGU-3', antisense: 5'-UGAGGGGCCUCAGACCGAGCUUU-3'; miR-93-5p mimics: sense: 5'-CAAAGUGCUGUUCGUGCAGGUAG-3', antisense: 5'-ACCUGCACGAACAGCACUUUGUU-3'; miR-21-5p mimics: sense: 5'-UAGCUUAUCAGACUGAUGUUGA-3', antisense: 5'-AACAUCAGUCUGAUAAGCUAUU-3'; miR-3656 mimics: sense: 5'-GCGGGUGCGGGGGUGGGC-3', antisense: 5'-CCACCCCCGCACCCGCUU-3'; mimics negative control: sense: 5'-UUCUCCGAACGUGUCACGUTT-3', antisense: 5'-ACGUGACACGUUCGGAGAATT-3'; miR-423-3p inhibitor: 5'-ACUGAGGGGCCUCAGACCGAGCU-3'; miRNA inhibitor negative control: 5'-CAGUACUUUUGUGUAGUACAA-3'; Beclin1 knockdown RNA: sense: 5’-GCUGCCGUUAUACUGUUCUTT-3', antisense: 5'-AGAACAGUAUAACGGCAGCTT-3'

**EdU (5-ethynyl-2′-deoxyuridine) cell proliferation assay**

The EdU cell proliferation assay (Rib-bio; Guangzhou, China) was used to detect the proliferation rate of NHAs. EdU was added to the medium for 2 h at 37°C before cells were fixed with 4% polyformaldehyde for 30 min and incubated with 2 mg/ml glycine for 5 min. The incubated cells were permeabilized with 0.5% Triton X-100 for 10 min. The permeabilized cells were stained with Apollo® reagent for 30 min and Hoechst for 30 min. The fluorescent cells were observed with a fluorescence microscope.

**Transwell assay**

The NHAs were plated in the top chamber of Transwell cells with serum-free medium, and the bottom chamber was filled with DMEM with 10% FBS. After 24-48 h, the cells were fixed with 4% paraformaldehyde and stained with 0.1% crystal violet. The migrating cells were captured under the bright-field microscope and counted by ImageJ.

**Cell proliferation and viability**

P3#GBM were seeded in 96-wells plates at the density of 5.0 x 10^3^ /well. After 24h, P3#GBM were treated with GDC-0879 (1.25-320μM, MedChemExpress, Monmouth Junction, NJ, USA), 15d-PGJ2 (0.6-160μM, MedChemExpress, Monmouth Junction, NJ, USA), linsitinib (0.6-160μM, MedChemExpress, Monmouth Junction, NJ, USA) for 48h. Then 10μL cell counting kit 8 (CCK-8, Yeasen Biotechnology, Shanghai, China) was added to the medium in each well. The absorbance of medium was measured at 450 nm by Multiskan FC Microplate Photometer (Thermo Fisher Scientific, Waltham, MA, USA) after incubating in 37℃ for 2h. The IC50 was calculated with GraphPad Prism 8 software (GraphPad Software Inc., La Jolla, CA, USA).

**MiRNA array**

Exosomes derived from U251 and P3#GBM cells were stored at -80°C and shipped on dry ice to LC-Bio (Hangzhou, China), which finished the miRNA array.

**qPCR array**

NHAs in 6-well plates growing to 70%-80% density were washed in 1 ml of TRIzol (Invitrogen, Carlsbad, CA, USA) and transported to 1.5 ml Eppendorf tubes without RNase. The samples were stored at -80°C and shipped on dry ice to WcGene (Shanghai, China), which finished the qPCR array.

**RNA-seq**

NHAs in 6-well plates were washed in 1 ml of TRIzol (Invitrogen, Carlsbad, CA, USA) and transported to 1.5 ml Eppendorf tubes without RNase. The samples were stored at -80°C and shipped on dry ice to CapitalBio (Beijing, China), which finished the RNA-seq array.

**Intracranial animal model**

Four-week-old male BALB/c nude mice (male) were purchased from GemPharmatech Co., Ltd. (Nanjing, China). To test the glioma-astrocytes interactions in vivo, NHAs were treated with PBS, N-GDEs, or H-GDEs for 48 h. The treated NHAs were mixed with luciferase-labeled U251 and P3#GBM cells and microinjected into the parietal lobe of nude mice. The exosomes or PBS were injected into three groups of mice through the caudal vein every three days respectively. The tumor volume was quantitively detected through the different intensities of bioluminescence using the IVIS Lumina Series III (PerkinElmer, Waltham, MA, USA) every week. After 4 weeks, three mice in every group were randomly selected and euthanized to obtain the xenograft samples for subsequent experiments. Other mice were observed until death for survival analysis.

In the other part of the in vivo experiment, we used luciferase-labeled P3#GBM cells transfected with lenti-ov-miR-423-3p or lenti-miR-control to establish the animal models. Both kinds of GBM cells were mixed with NHAs and transplanted into the parietal lobe of nude mice. The detection of the tumor volume was the same as the above in vivo experiment. After 4 weeks, three mice in every group were randomly selected and euthanized to obtain brain samples for subsequent experiments. Other mice were observed until death for survival analysis.

All in vivo experiments were reviewed and approved by the Animal Care and Use Committee of Qilu Hospital of Shandong University. The average diameter of tumors in adult mice did not exceed 20mm, which was permitted by Animal Care and Use Committee of Qilu Hospital of Shandong University. All in vivo experiments and tumor size conform to all relevant regulatory standards.

**Immunohistochemistry (IHC)**

The xenograft samples were fixed with 4% paraformaldehyde and embedded in paraffin for subsequent experiments. Tissue samples were hydrated with ethanol, dimethylbenzene, reacted with primary antibodies against GFAP (Proteintech, Chicago, IL, USA) and Ki67 (Servicebio, Wuhan, Hubei, China) at 4°C overnight. Primary antibody-treated tissue samples were conjugated with secondary antibody and reacted with 3,3′-diaminobenzidine (DAB) for visualization. Then, the samples were stained with hematoxylin^1^. The images were taken by a Leica DM 2500 microscope.

**Non-contact cell co-culture system**

The 12 mm Transwell with 0.4 μm pore polyester membrane inserts (Corning Inc. San Francisco, CA, USA) were used to perform experiment. P3#GBM cells were cultured in inserts with NHAs seeded in lower chamber for 5 days. Subsequently, P3#GBM cells were collected and reseeded for further analysis. The medium used for both P3#GBM and NHAs, as mentioned earlier, was mixed to support the co-culture system.

**Ethics statement**

All experimental procedures were authorized by the Ethics Committee of Qilu Hospital (Jinan, China) and conducted in compliance with the associated guidelines and regulations. All patients have obtained written informed consent.

**Supplemental figure legends**

**Figure.S1 A.** Immunofluorescence staining for PKH67(green) to detect absorption of exosomes in NHAs, cytoskeleton was stained by Phalloidin(red) to show the cell structure. Cell nucleus was stained with DAPI (blue) (scale bar,25μm). **B.** NTA analysis to detect the concentration and diameters of exosomes derived from U251 and P3#GBM under normoxia and hypoxia circumstance respectively.

**Figure.S2 A.** Western blot analysis detecting the expression of becn1 and GAPDH in NHAs treated with siNC and siBECN1 was used to confirm the knocking down efficiency of siBECN. **B.** Immunofluorescence staining for MAP1LC3B(green) to detect the level of autophagy in NHAs treated with PBS, N-GDEs and H-GDEs derived from U251 for 48h, cell nuclei were stained by DAPI (blue) (scale bar,25μm). **C, D.** The statistic results of the relative immunofluorescence intensity in the images shown in figure1F-1H and figureS2B. (*P < 0.05; **P < 0.01; ***P < 0.001, n=3)

**Figure.S3 A.** qRT-PCR analysis to detect the expression of miR-423-3p in GDEs from U251 cells. **B.** A qRT-PCR analysis to detect the relative level of miR-423-3p in NHAs treated with miR-control and miR-423-3p mimics, and the statistic result was shown. **C.** qRT-PCR analysis to detect the relative expression level of PABPC1 and RAP2C in P3#GBM treated with miR-control and miR-423-3p mimic was carried on to verify the transfection of miR-423-3p. **D.** A qRT-PCR analysis to detect the relative expression level of miR-423-3p in the exosomes derived from P3#GBM treated with lenti-control and lenti-miR-423-3p+ was carried on to verify the transfection of miR-423-3p+ plasmid and the relative expression. **E.** A qRT-PCR analysis to detect the relative expression level of miR-423-3p in NHAs treated with inhibitor control and lenti-miR-423-3p inhibitor was carried on to verify the inhibition efficiency of miR-423-3p. (*P < 0.05; **P < 0.01; ***P < 0.001, n=3)

**Figure.S4 A-D.** The statistic results of the relative immunofluorescence intensity corresponding to figure3G, 3H, 3J and 3K. (*P < 0.05; **P < 0.01; ***P < 0.001, n=3) **E, F.** statistical analysis for the formation of autophagosomes depicted in figure3M. (*P < 0.05; **P < 0.01; ***P < 0.001, n=3)

**Figure.S5 A.** Statistical analysis of bioluminescence imaging of tumor-bearing mice treated with P3#GBM in conjunction with PBS-NHA, N-GDEs-NHA, H-GDEs-NHA on day 7. **B.** Statistical analysis of Ki67 IHC scores corresponding to the staining depicted in Figure 5D. **C.** The statistical evaluation of bioluminescence imaging data from mice on day 7 harboring tumors treated with P3#GBM alongside lenti-control-NHA or lenti-ov-miR-423-3p-NHA. (ns P≥0.05; *P < 0.05; **P < 0.01; ***P < 0.001, n=3).

**Figure.S6 A.** The GO enrichment analysis centered on the molecular function and biological process aspects, derived from transcriptome sequencing in NHAs treated with miR-control and miR-423-3p mimics.

**Figure.S7 A.** Survival curves for NHA to determine the IC50 values of GDC-0879 (1.25-320 μM) and linsitinib (0.6-160 μM) over 48 hours. **B.** Western blot analysis of protein extracts from NHAs treated with phosphate-buffered saline (PBS), H-GDEs and 15d-PGJ2, demonstrating the expression of GFAP and GAPDH. Relative GFAP levels of three replications are quantified via grayscale analysis with control set to 1. **C.** Representative images and statistical analysis showing the migration capacity of NHAs across Transwell chambers treated with PBS, H-GDEs, and 15d-PGJ2 (scale bar, 200 μm). **D.** EdU assay demonstrates NHAs proliferative response to PBS, H-GDEs, and 15d-PGJ2 treatments. Representative images and statistical analysis are shown (scale bar, 70μm)

1 Xu, J. *et al.* Cullin-7 (CUL7) is overexpressed in glioma cells and promotes tumorigenesis via NF-κB activation. *J Exp Clin Cancer Res* **39**, 59 (2020). <https://doi.org:10.1186/s13046-020-01553-7>
