# Supplementary figures and images for: Inhibition of hypoxic exosomal miR-423-3p decreases glioma progression by restricting autophagy in astrocytes
Source: Cell Death Dis. 2025 Apr 8;16(1):265. doi: 10.1038/s41419-025-07576-2 (PMC11978802; doi:10.1038/s41419-025-07576-2)

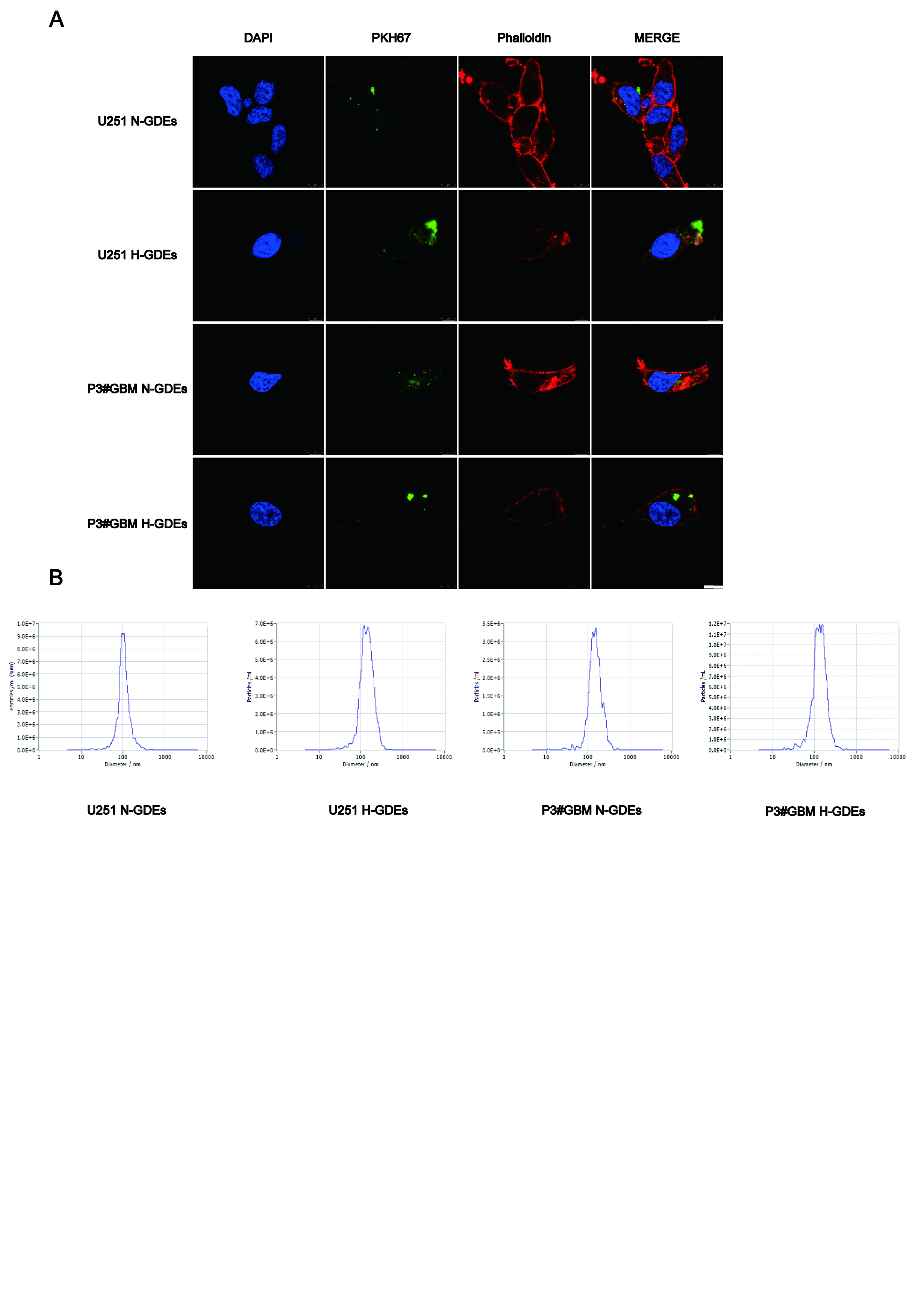

Supplement: Supplementary file 2 — Supplementary Figure 1 [file 41419_2025_7576_MOESM2_ESM.tif]

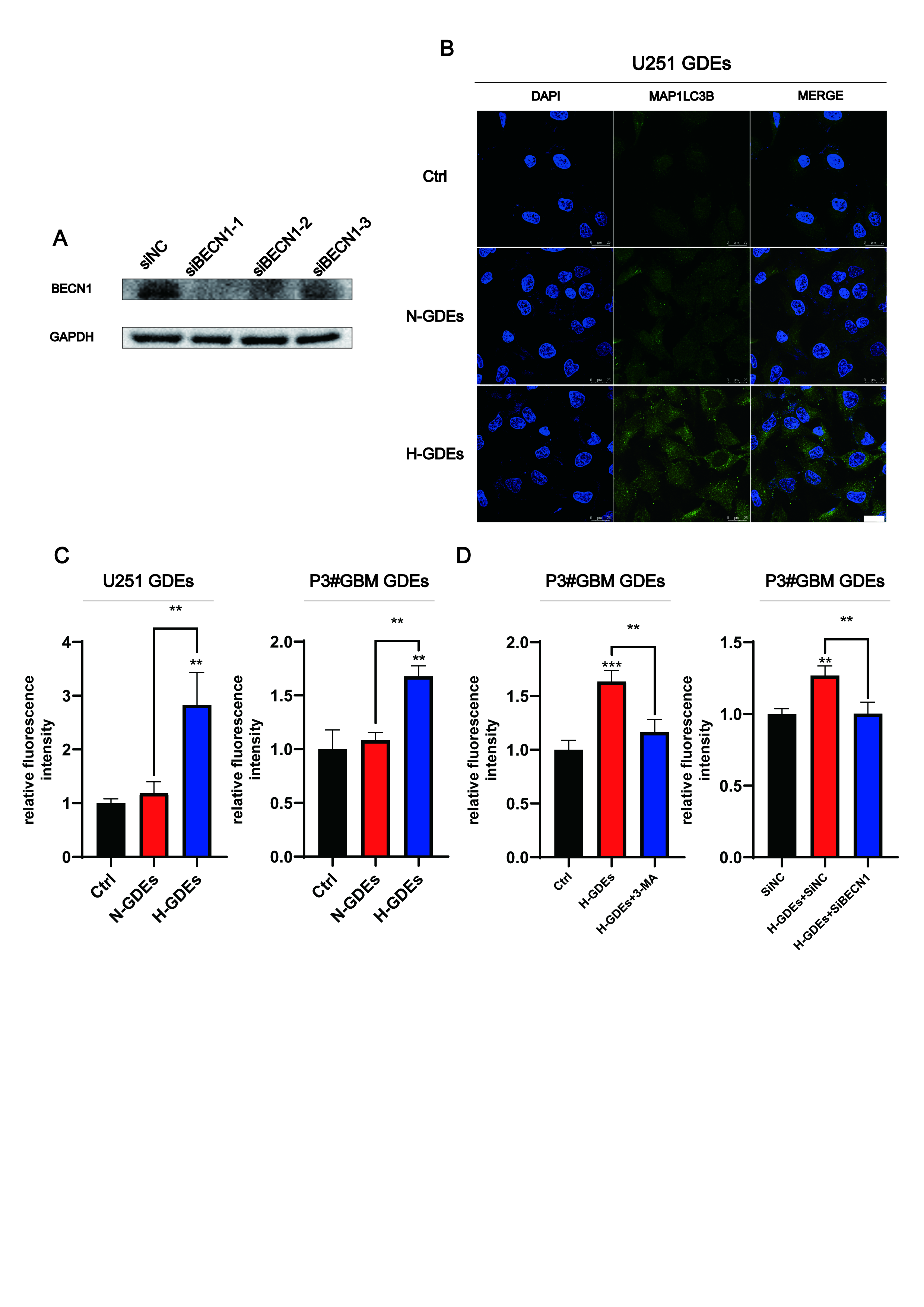

Supplement: Supplementary file 3 — Supplementary Figure 2 [file 41419_2025_7576_MOESM3_ESM.tif]

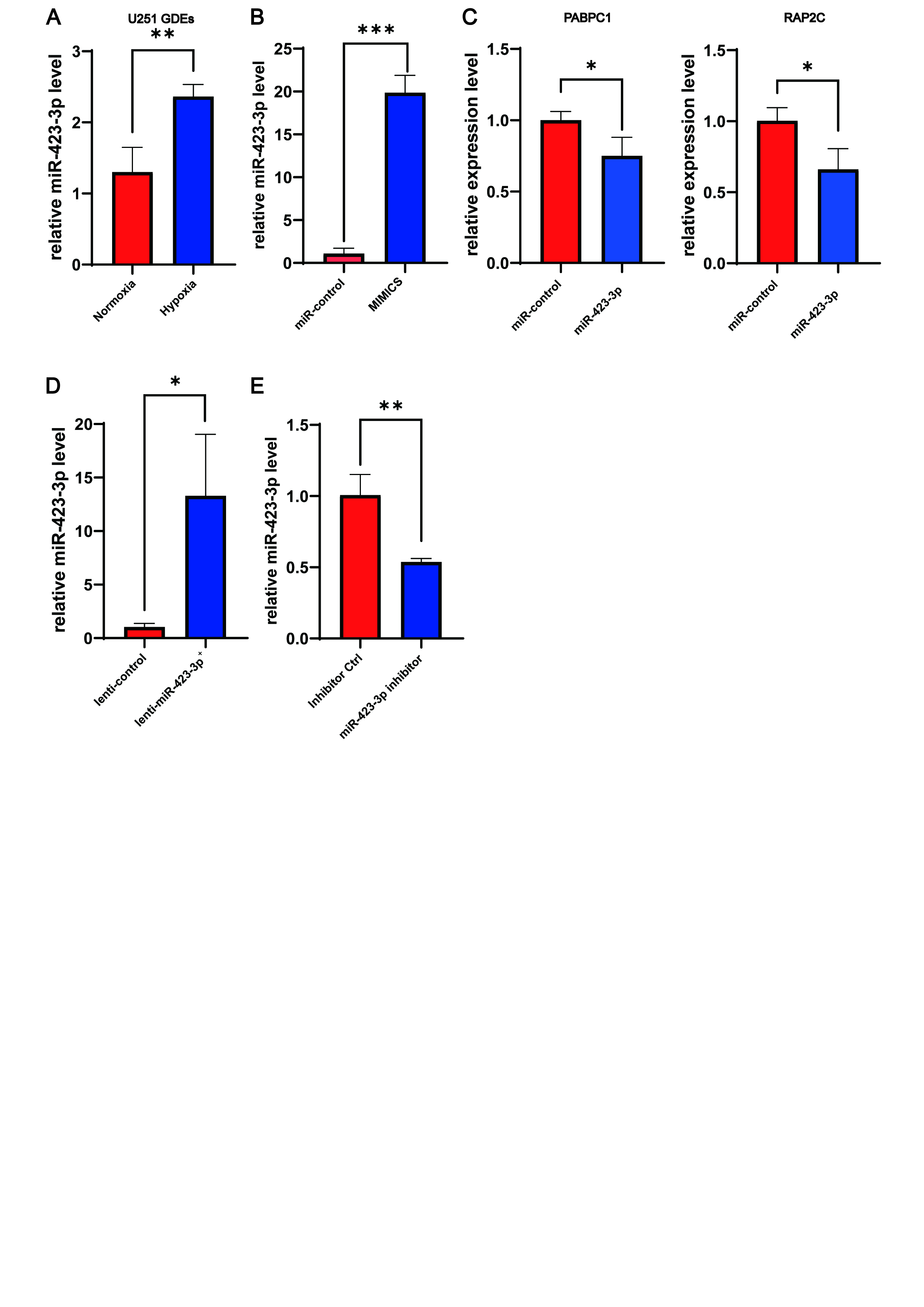

Supplement: Supplementary file 4 — Supplementary Figure 3 [file 41419_2025_7576_MOESM4_ESM.tif]

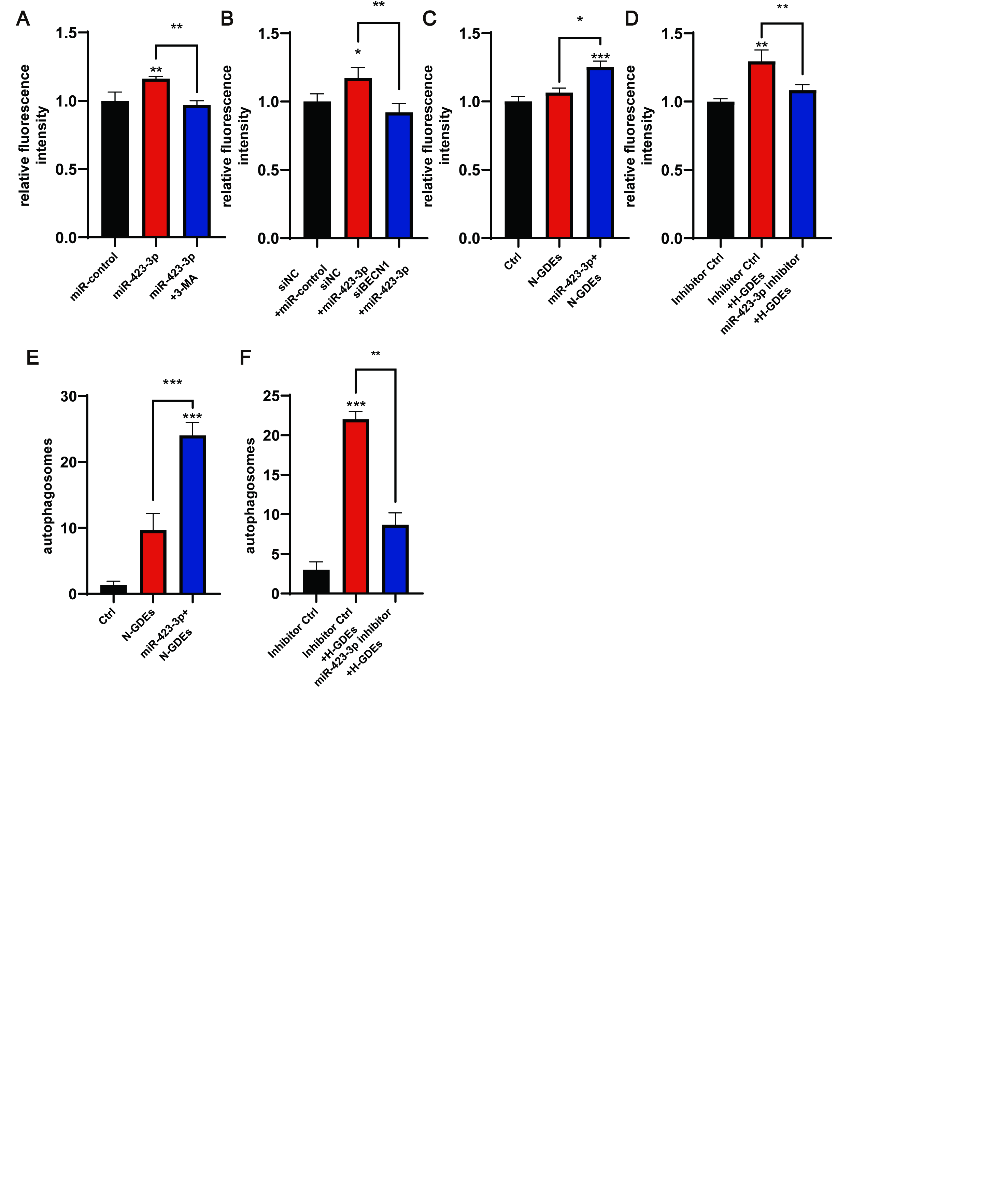

Supplement: Supplementary file 5 — Supplementary Figure 4 [file 41419_2025_7576_MOESM5_ESM.tif]

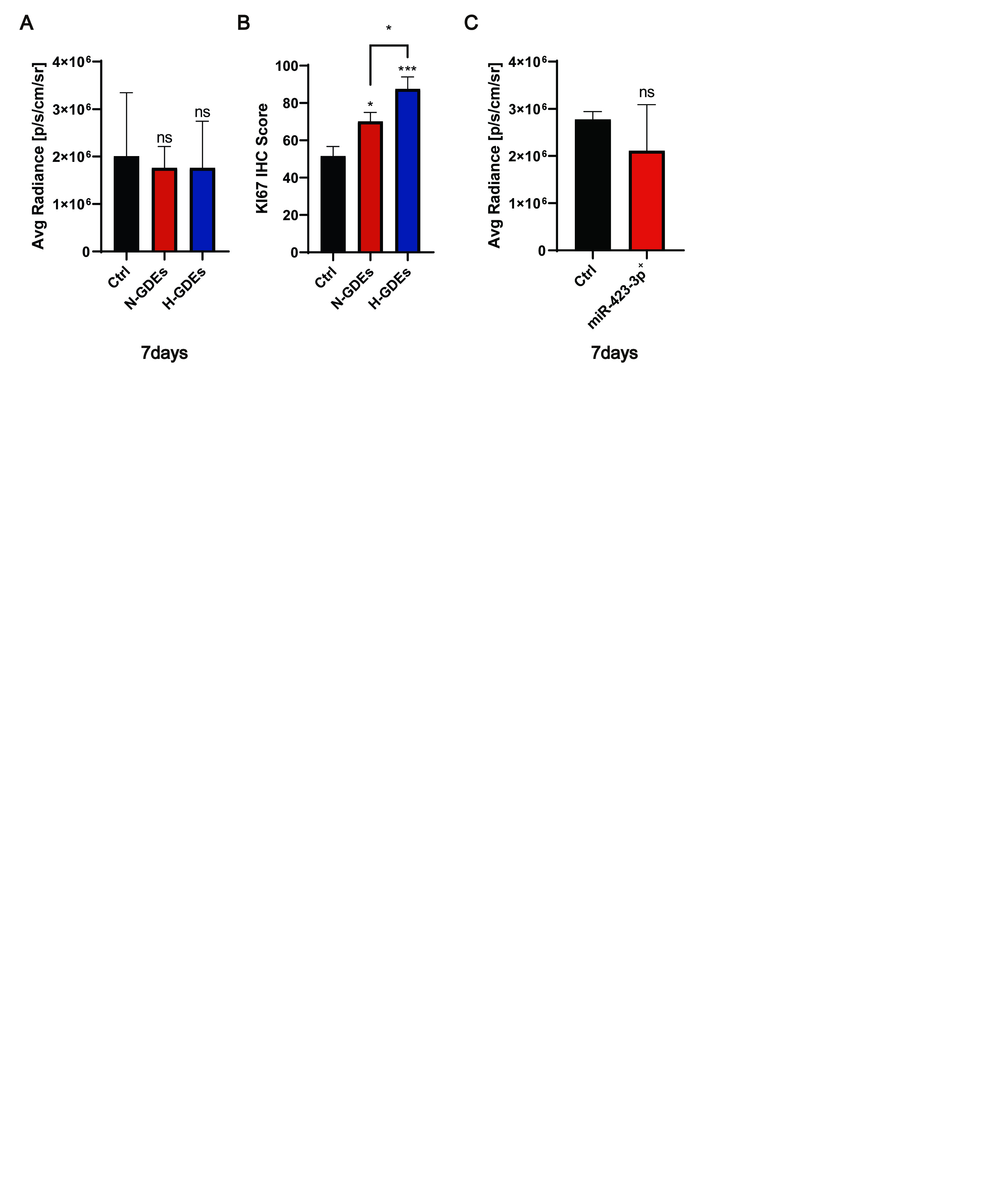

Supplement: Supplementary file 6 — Supplementary Figure 5 [file 41419_2025_7576_MOESM6_ESM.tif]

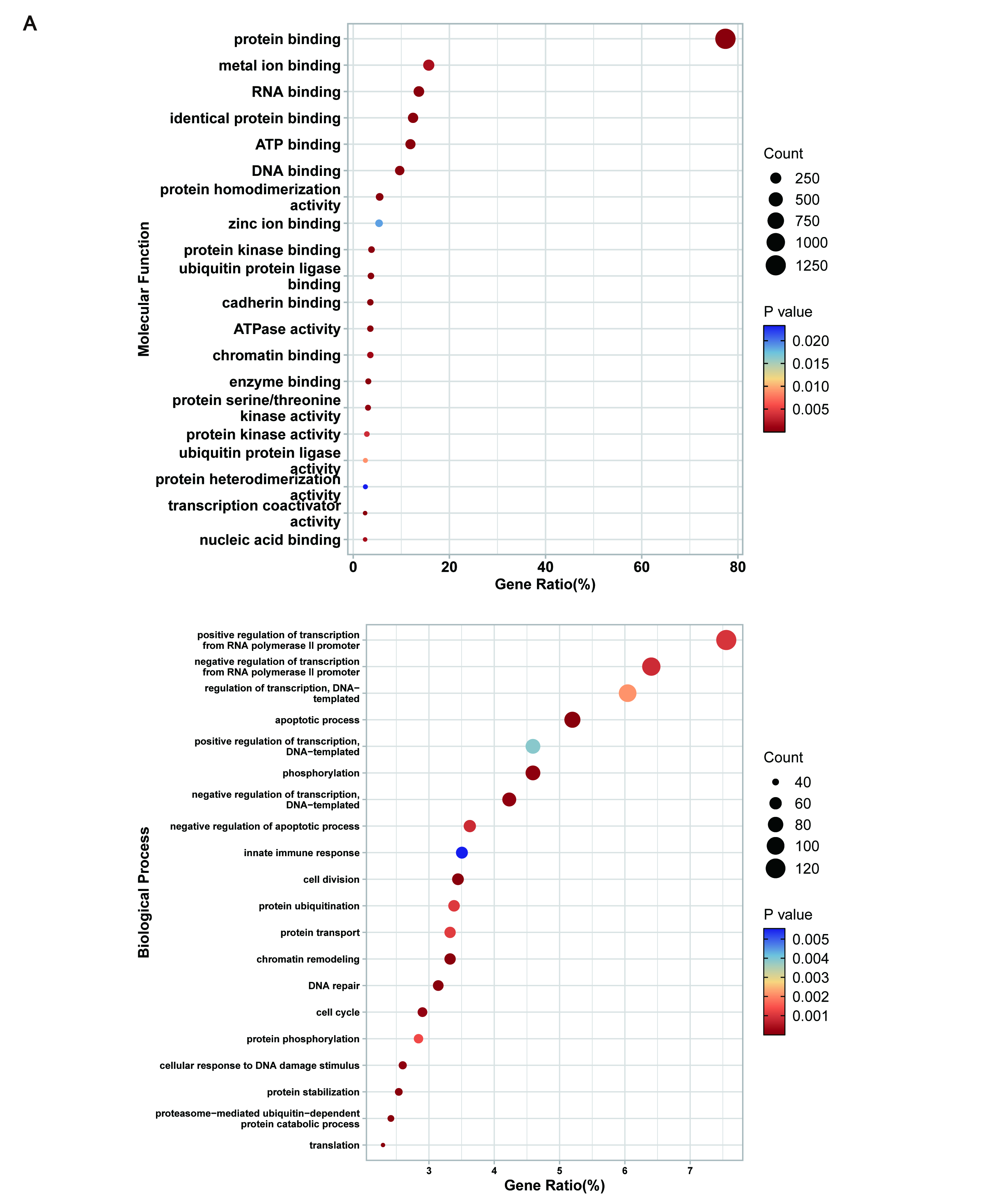

Supplement: Supplementary file 7 — Supplementary Figure 6 [file 41419_2025_7576_MOESM7_ESM.tif]

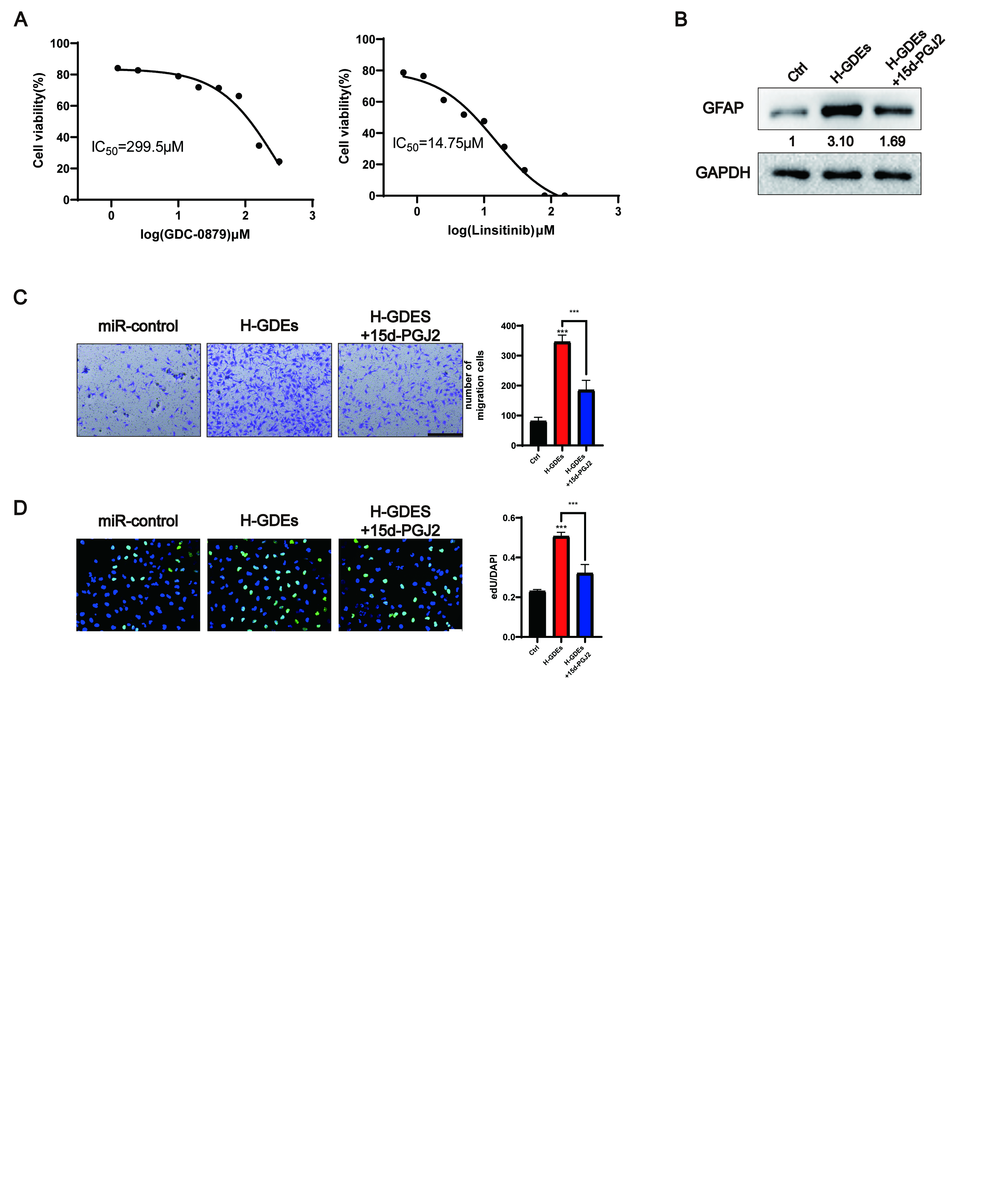

Supplement: Supplementary file 8 — Supplementary Figure 7 [file 41419_2025_7576_MOESM8_ESM.tif]

**Figure 1**





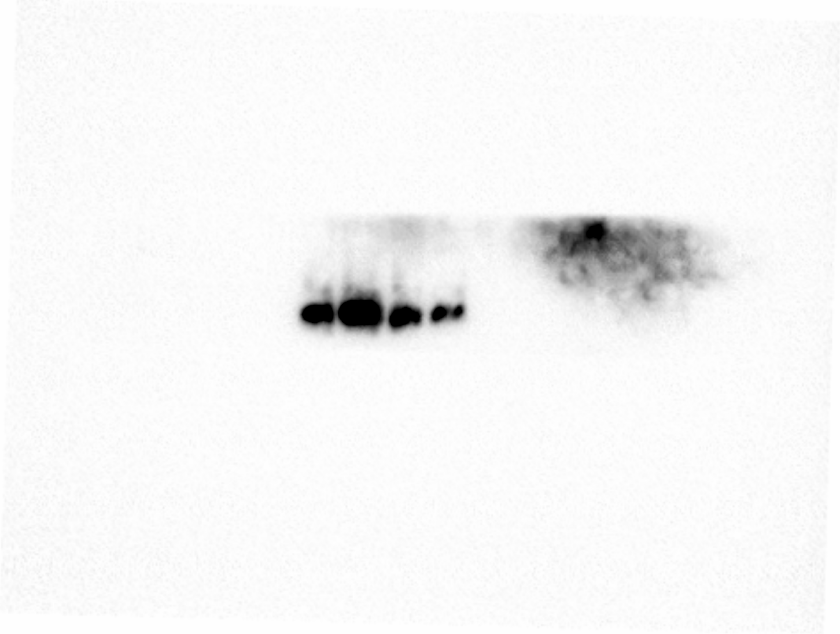


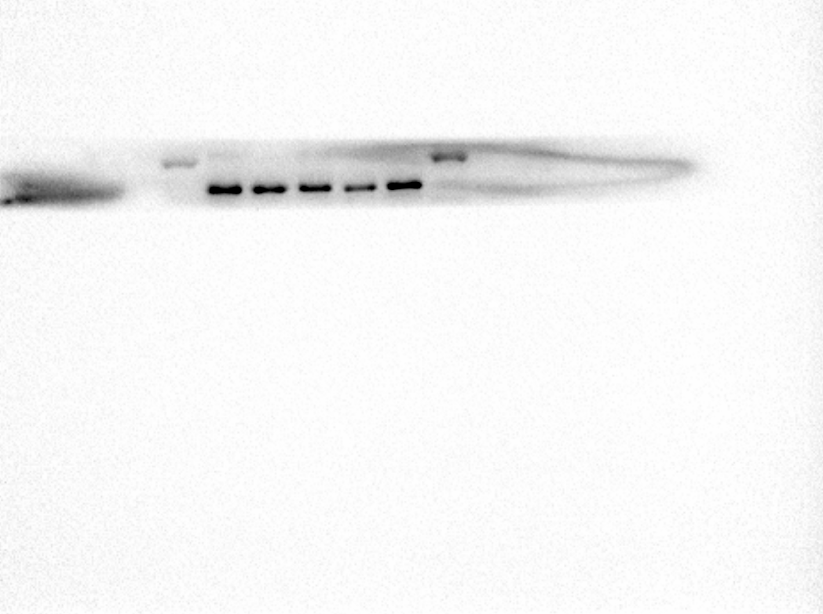


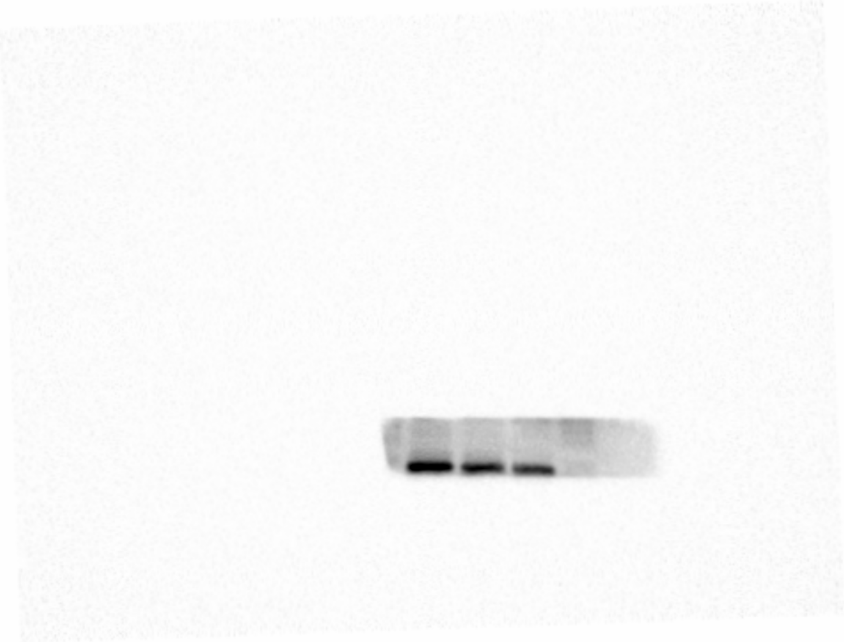

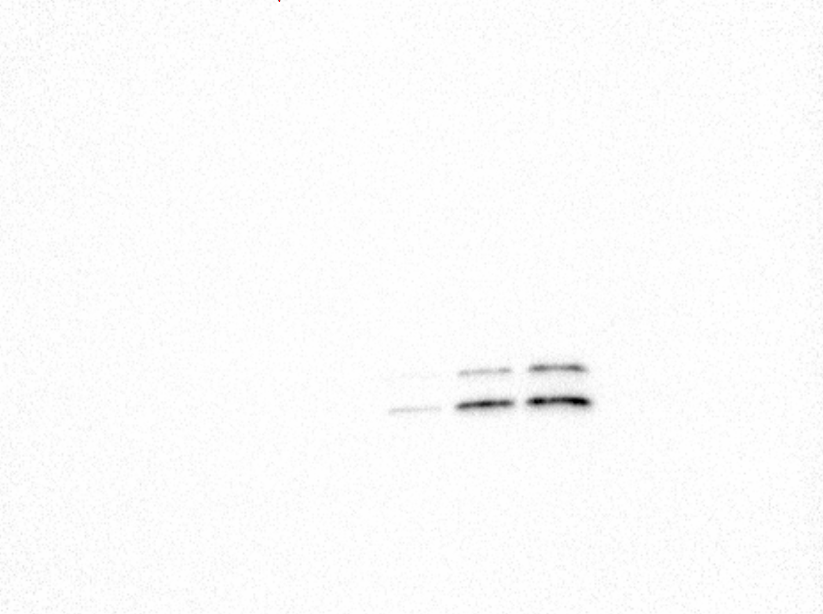

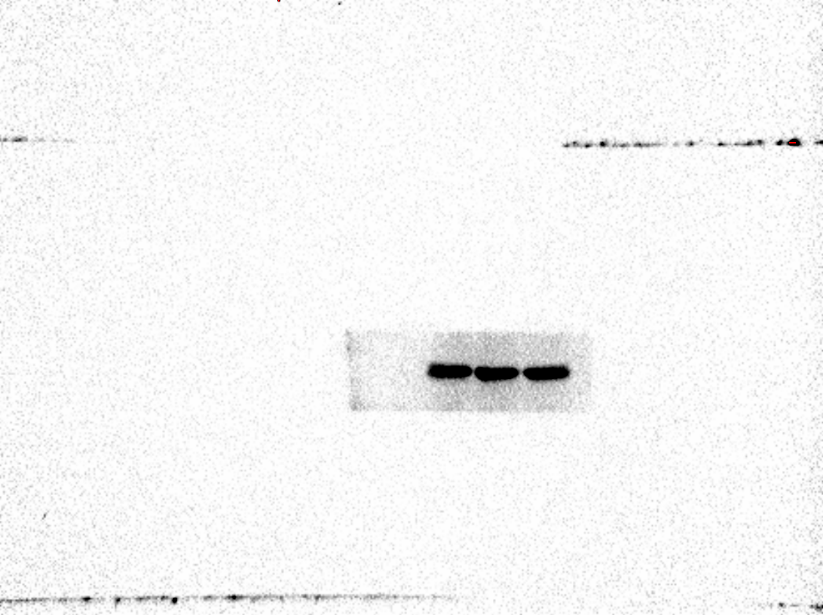


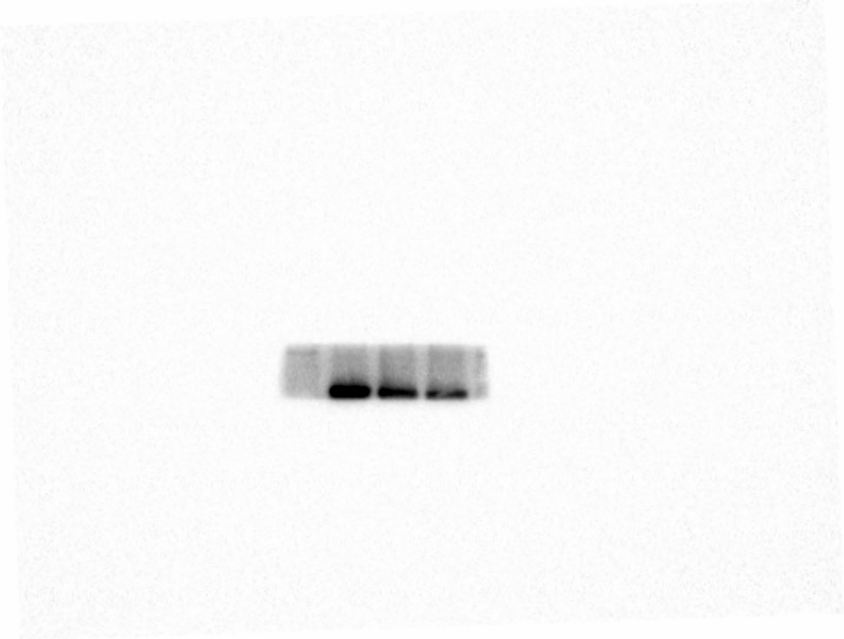

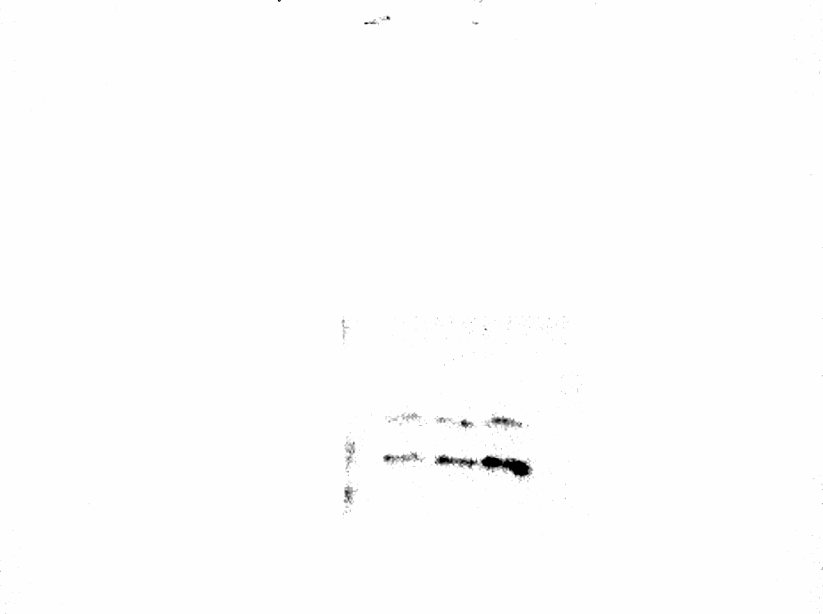

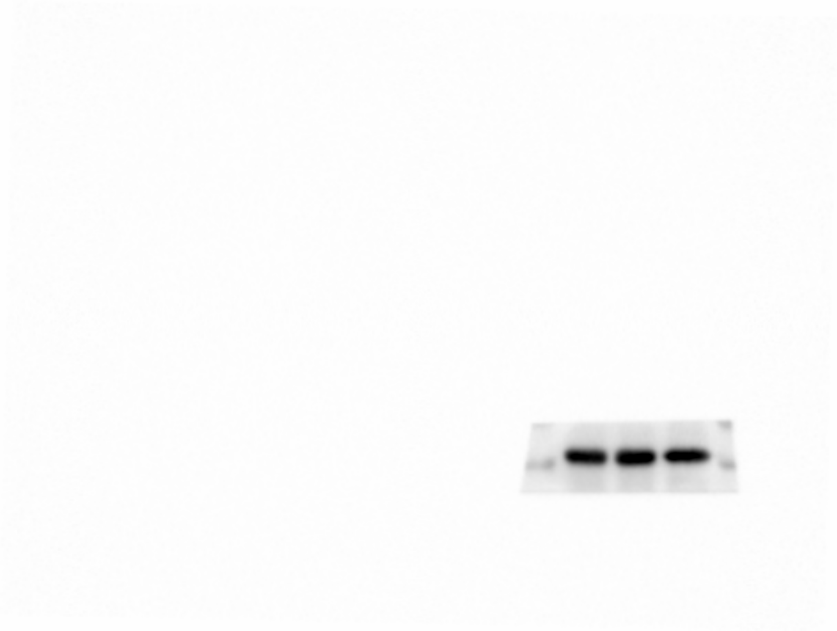


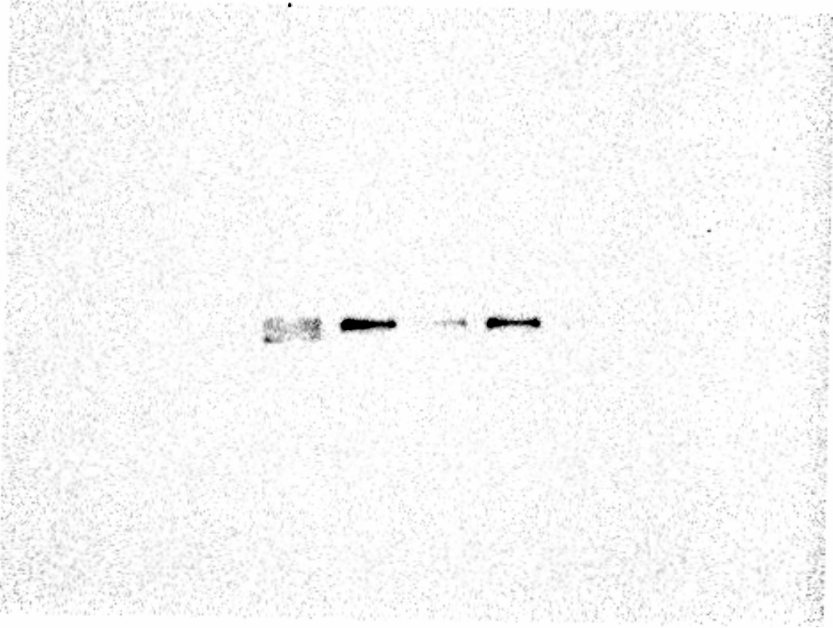


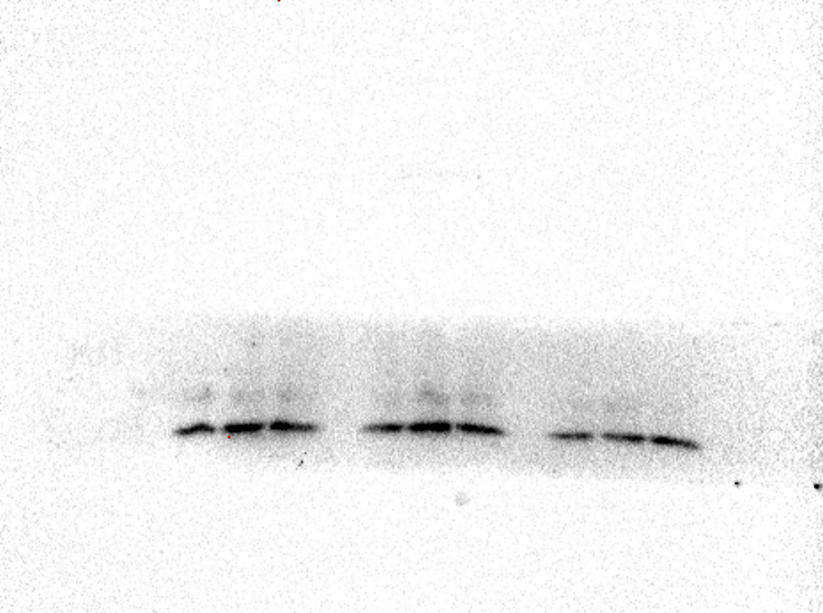


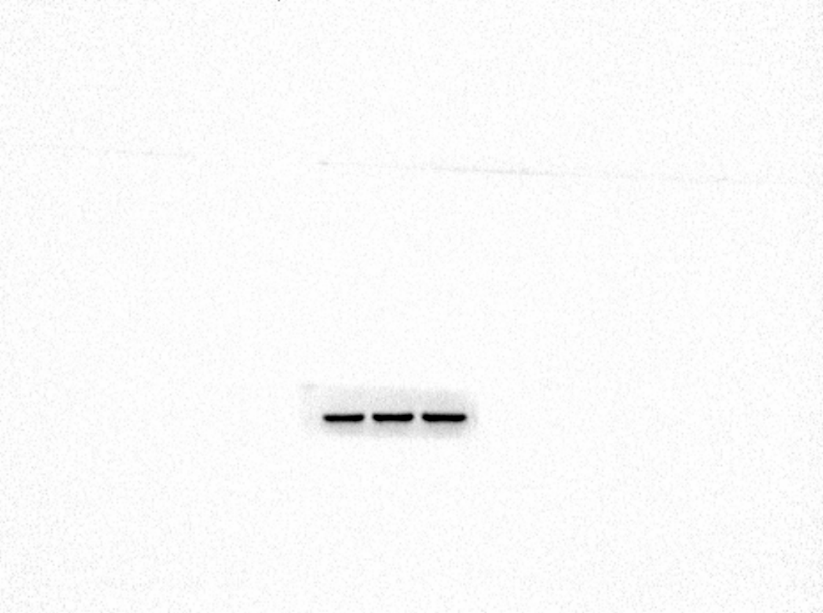


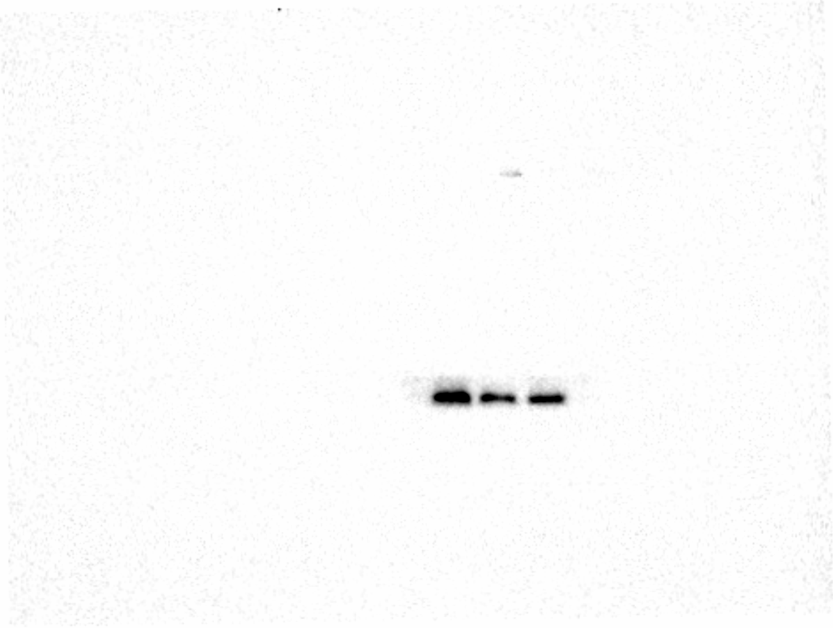


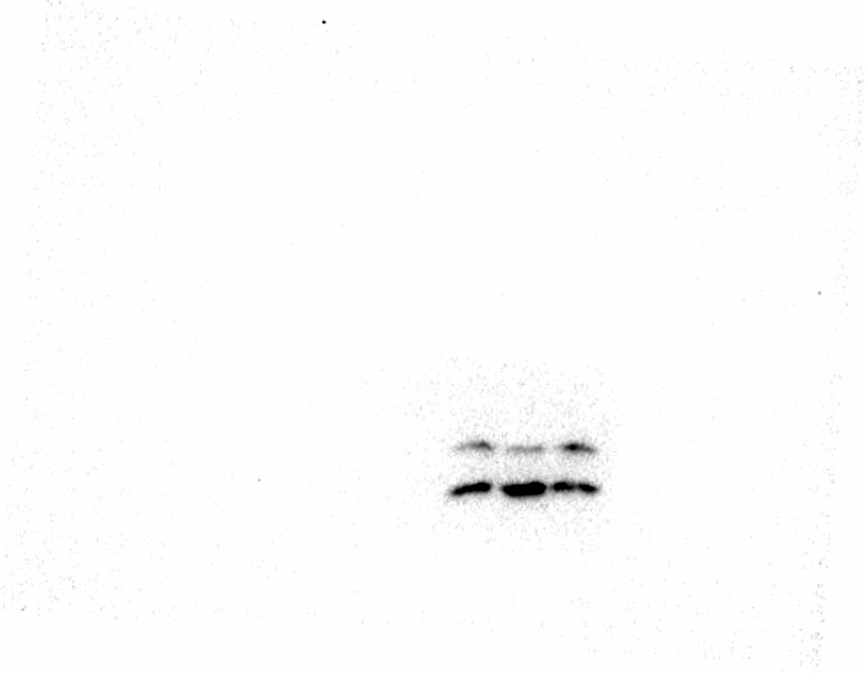


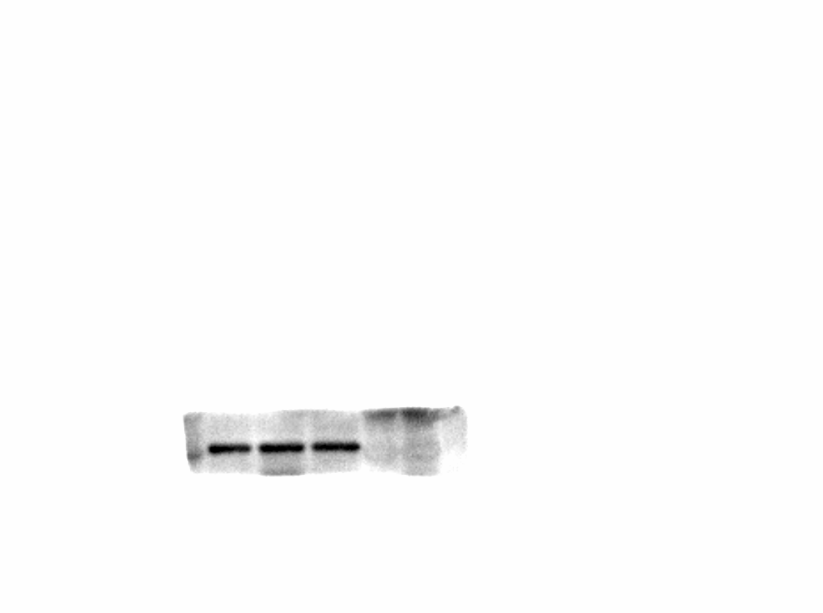


**Figure 2**


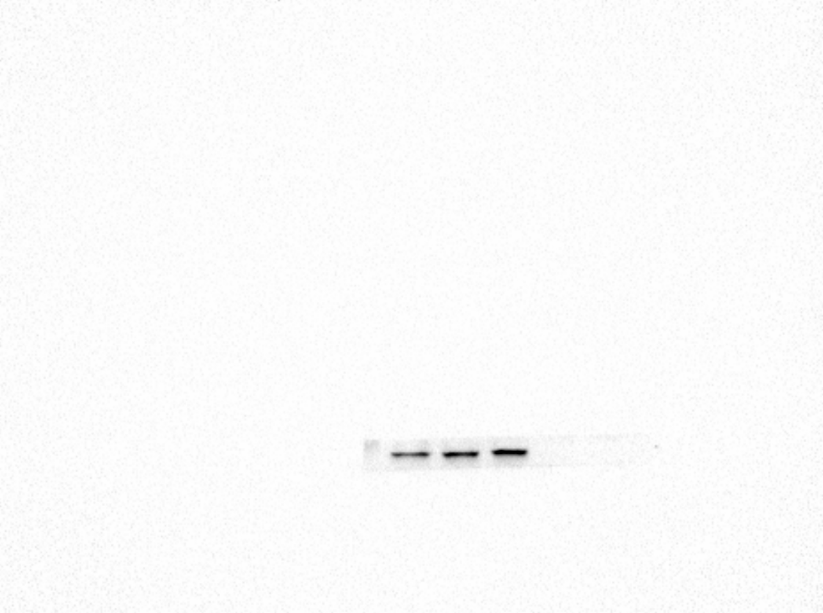


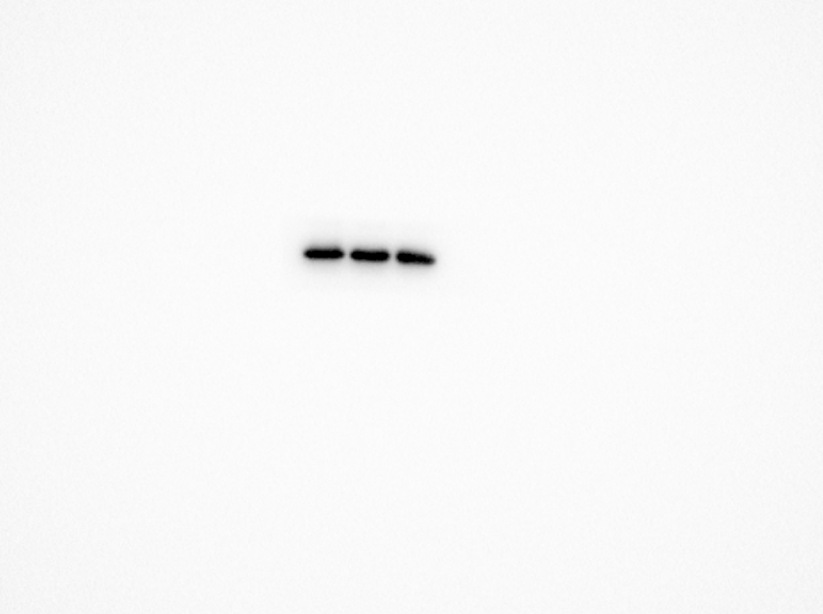


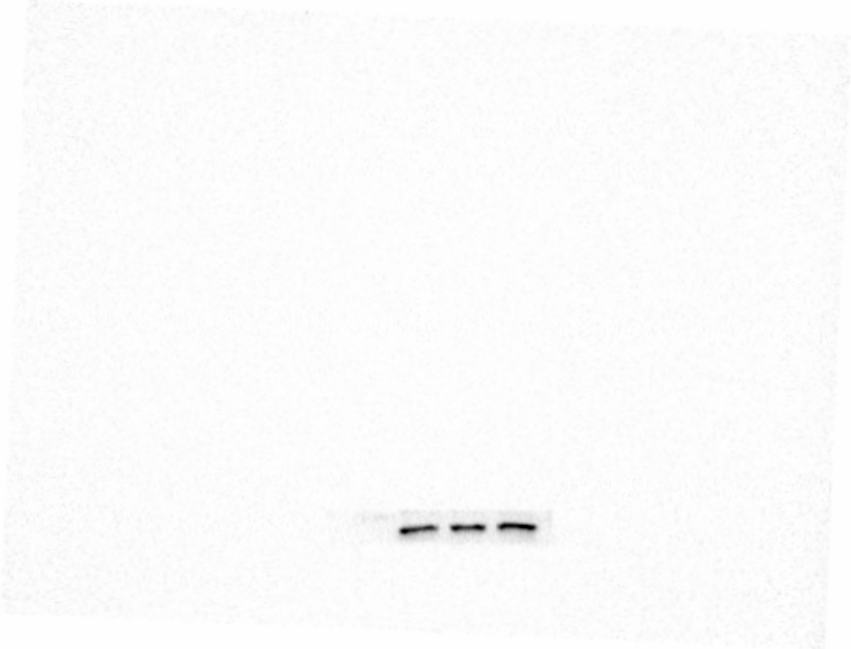


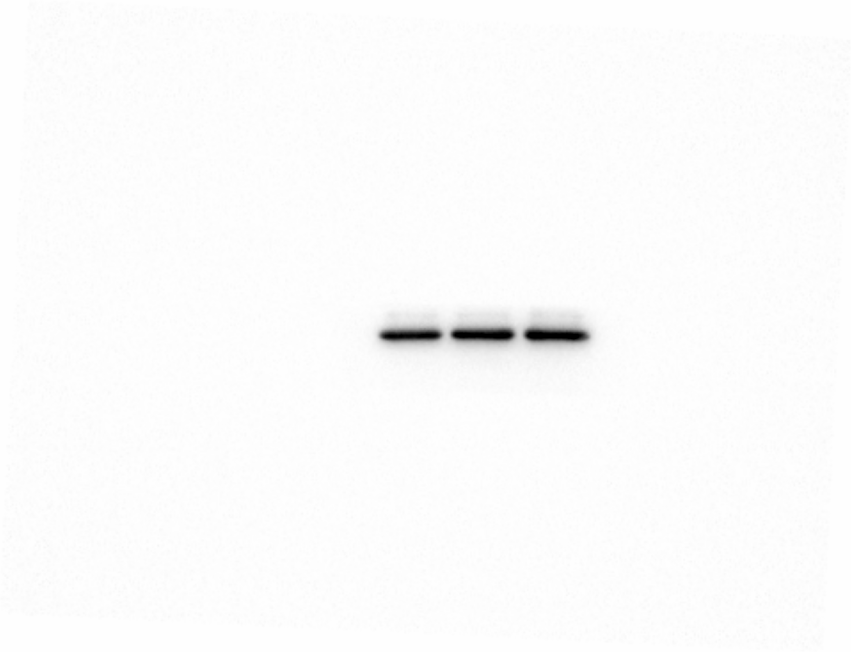


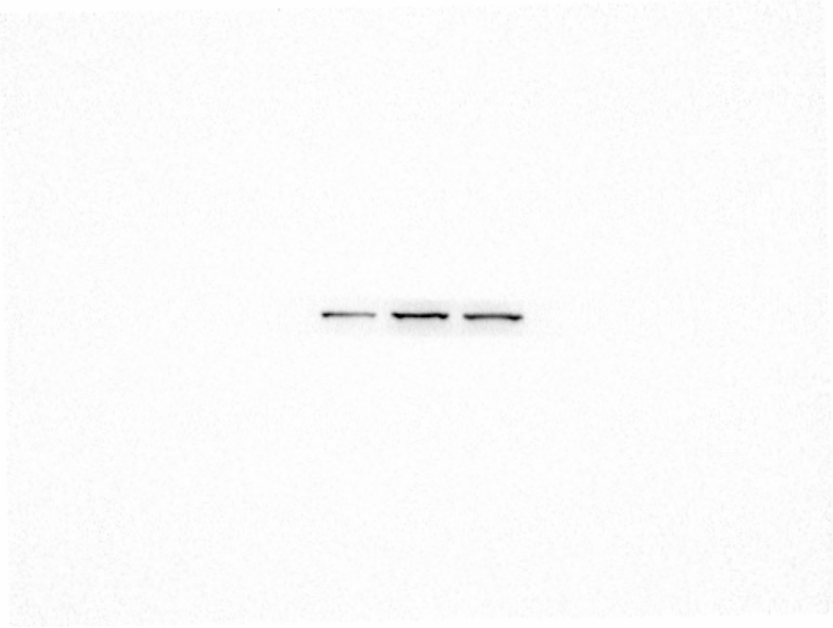

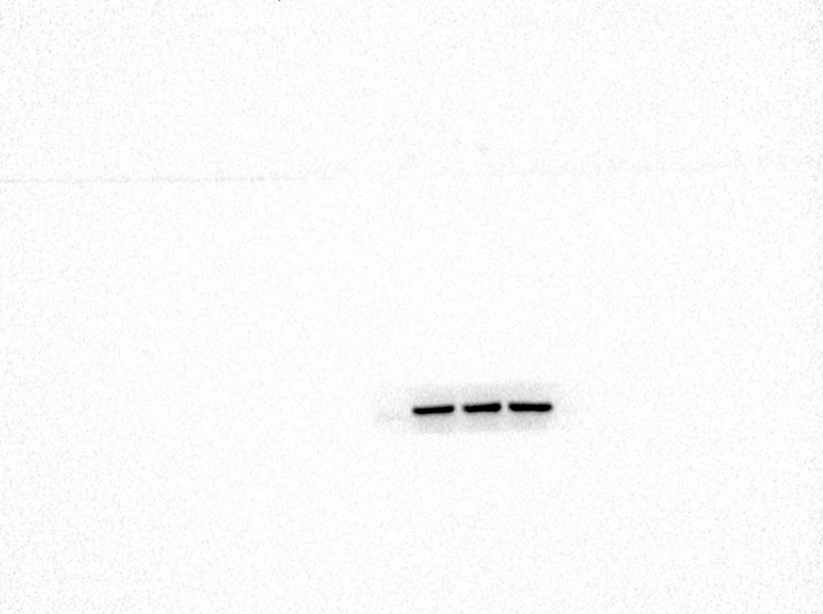

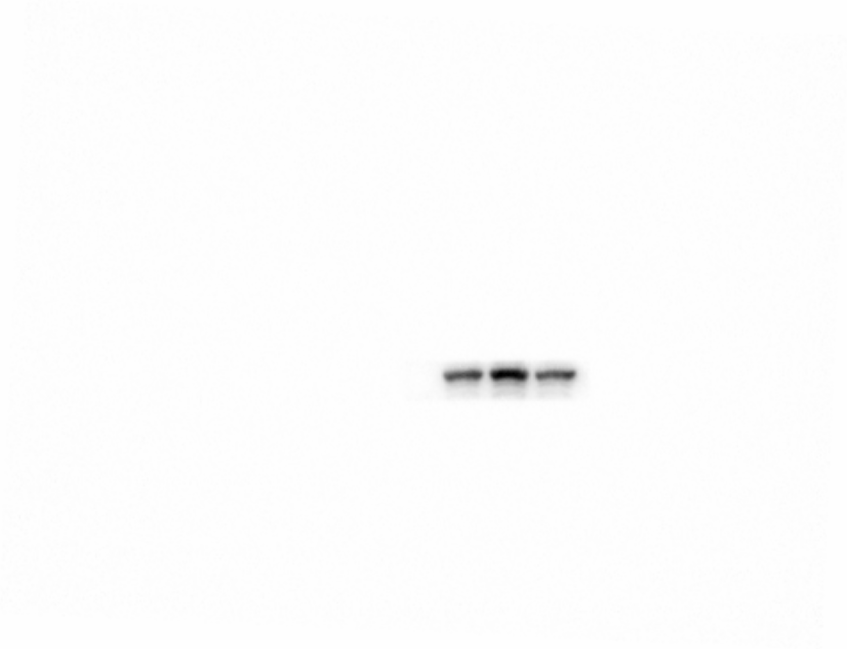


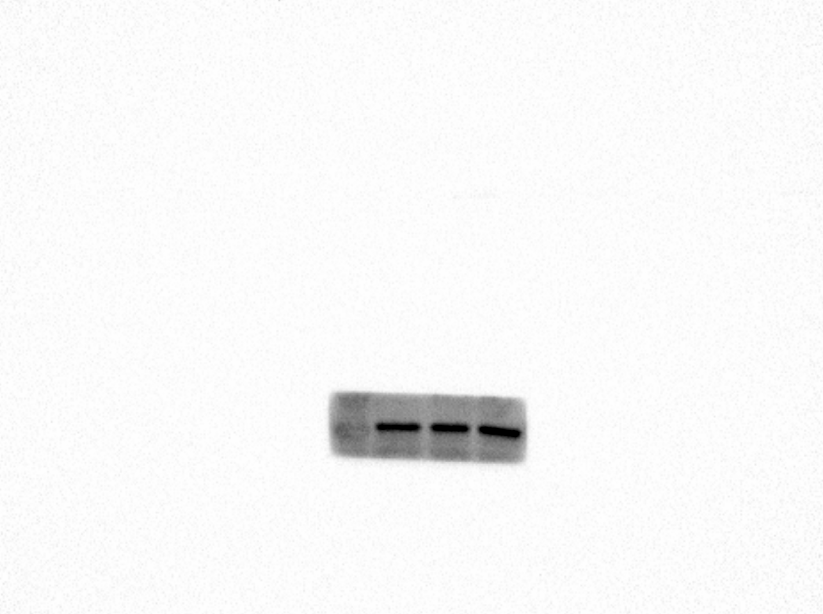


**Figure 3**


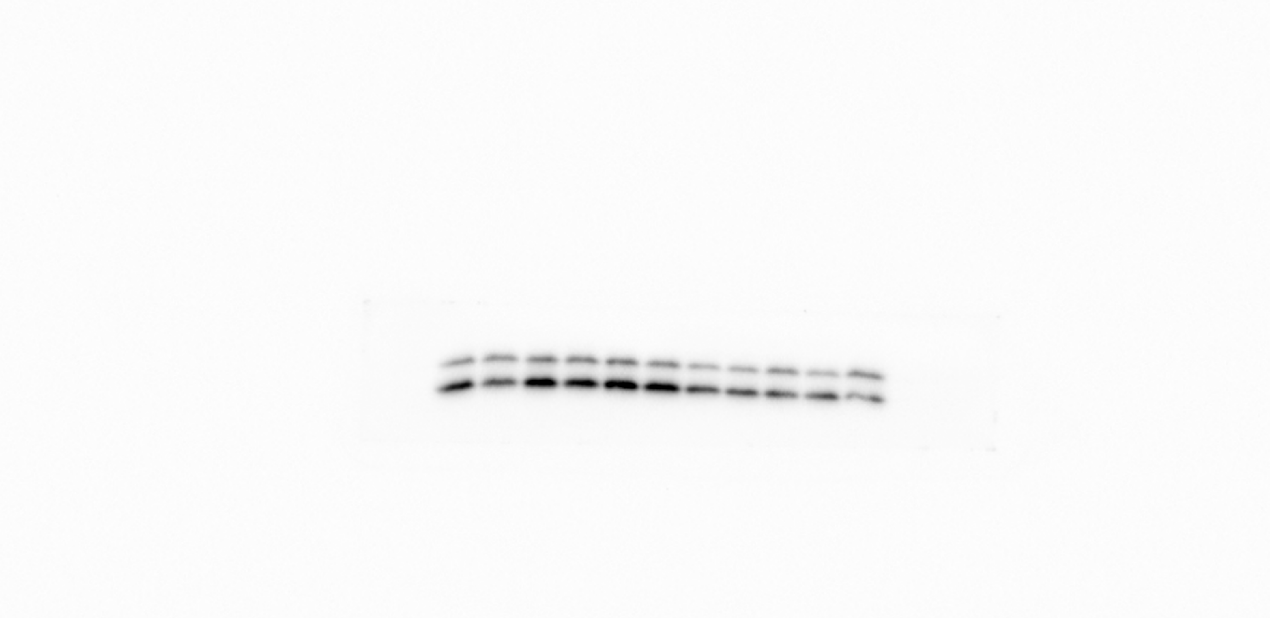

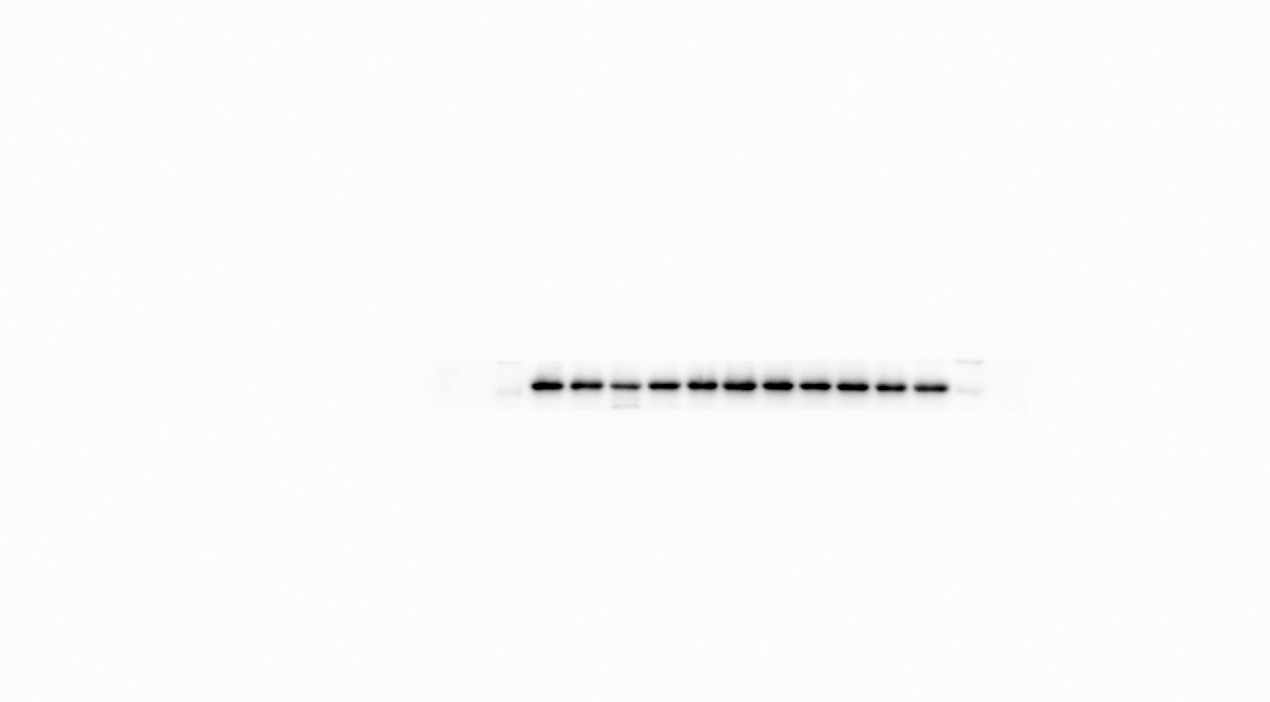


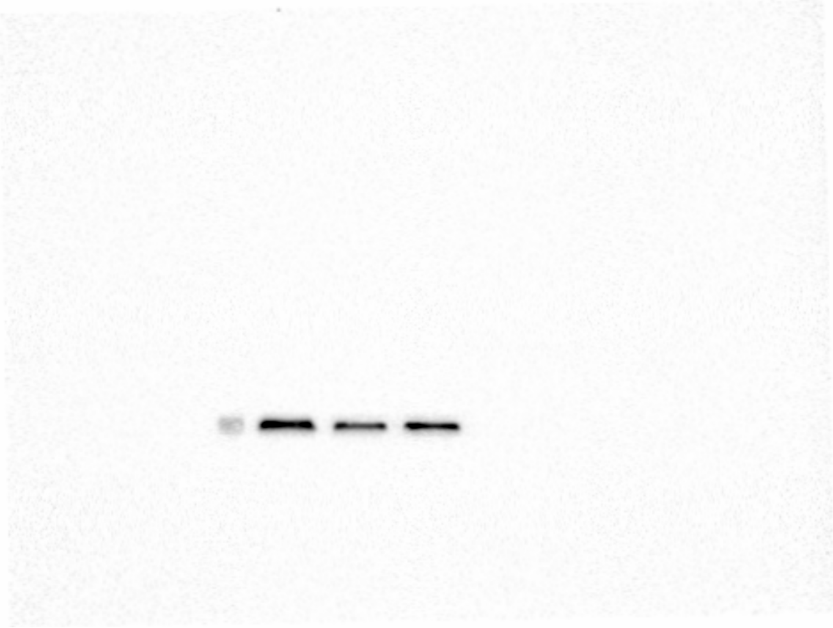

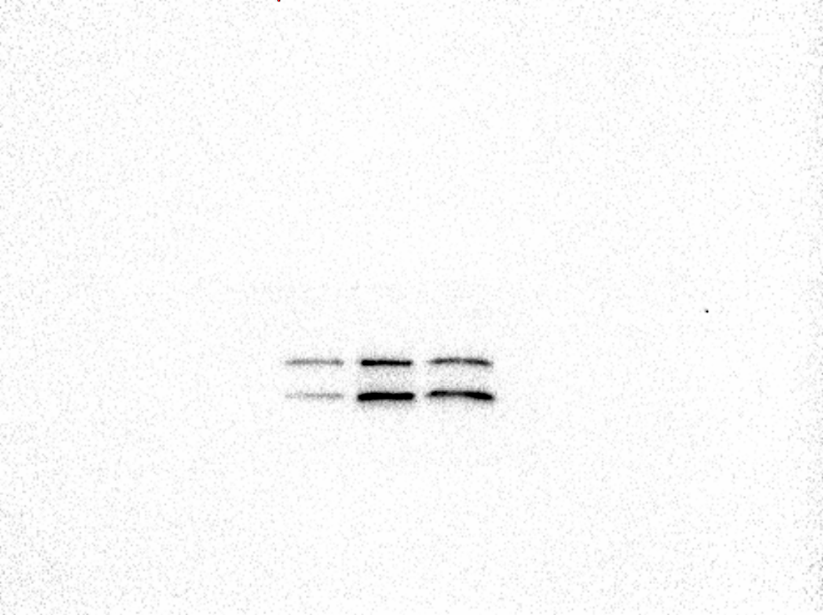

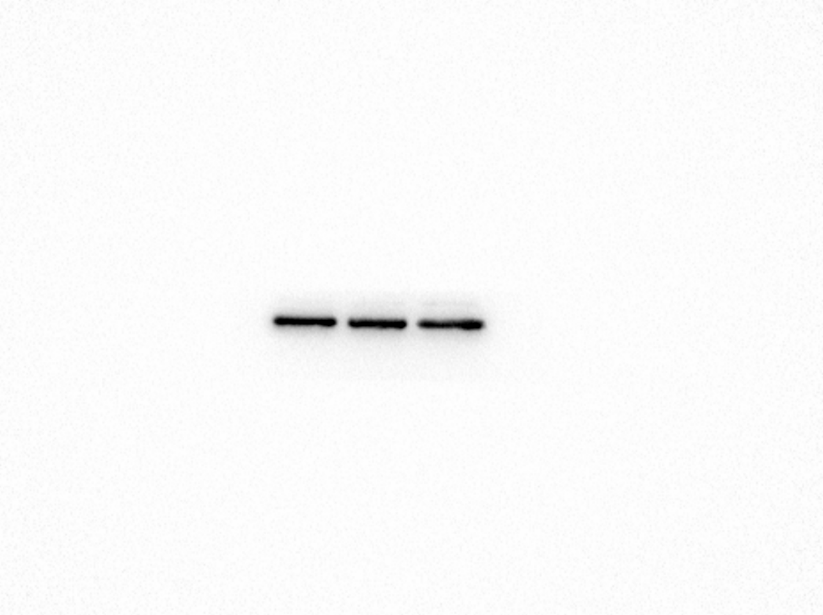


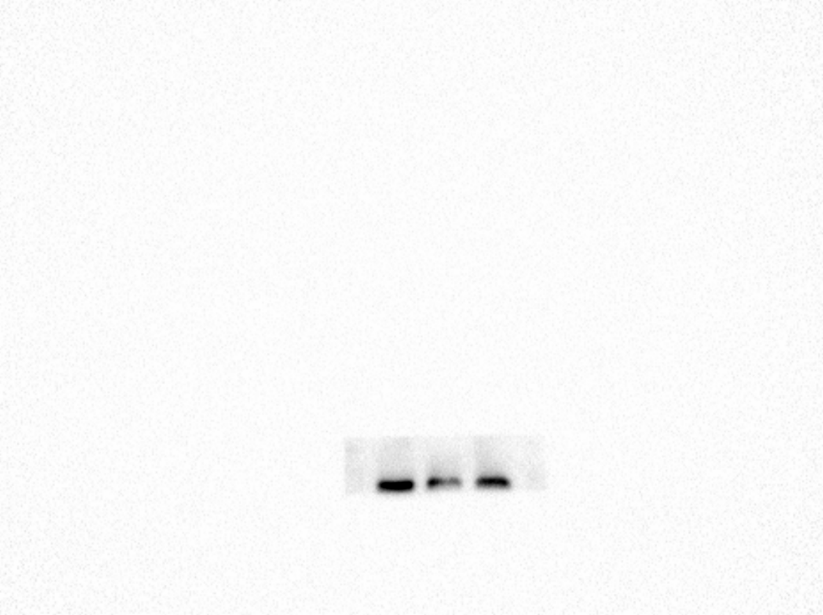

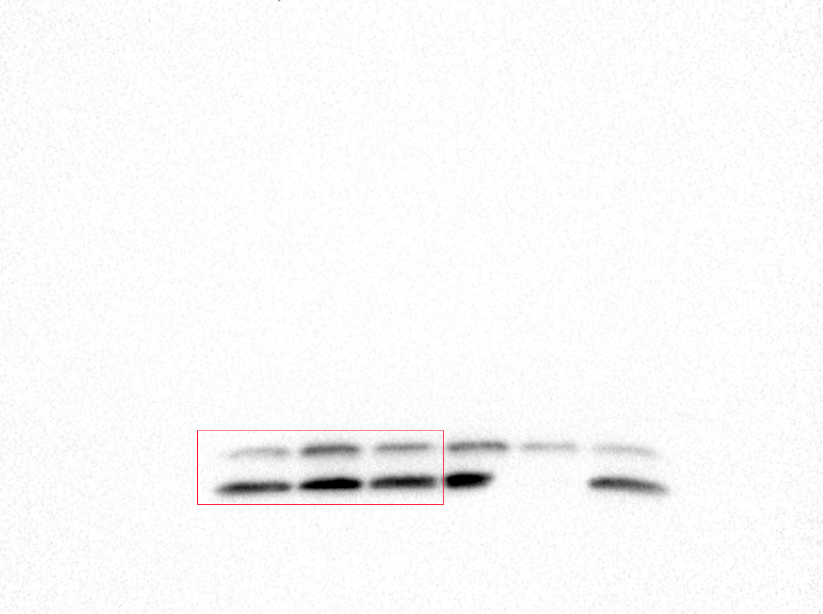

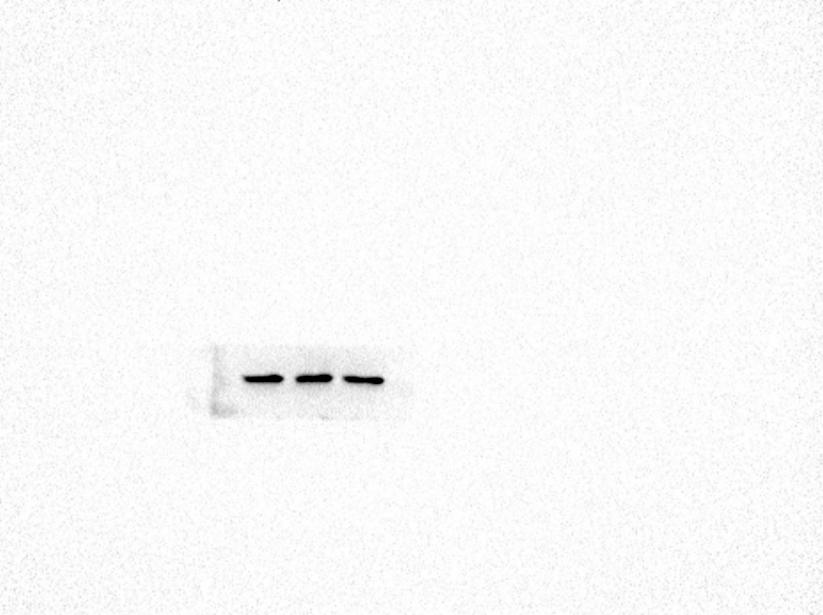

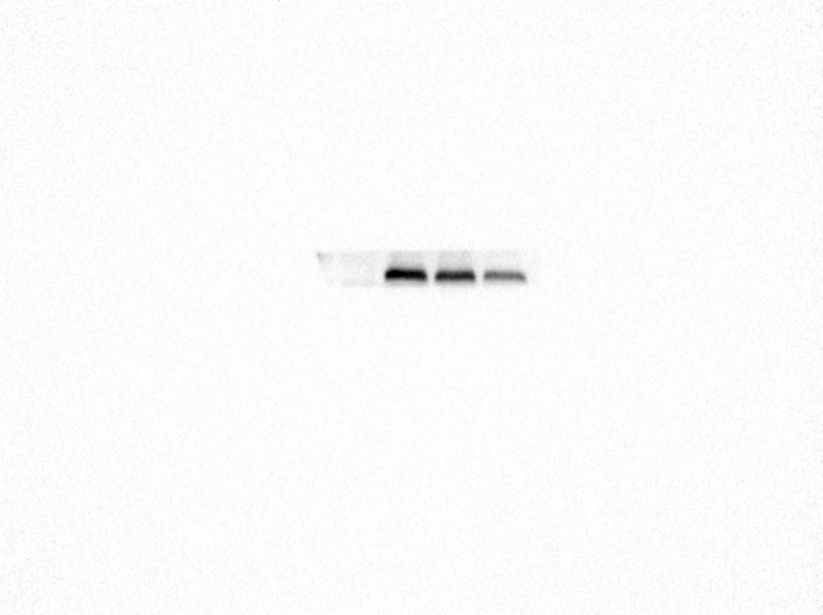

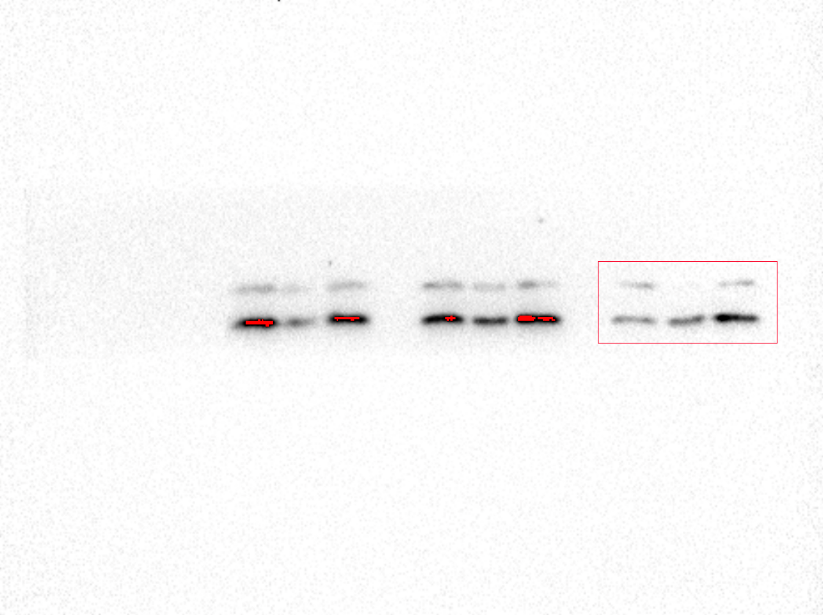

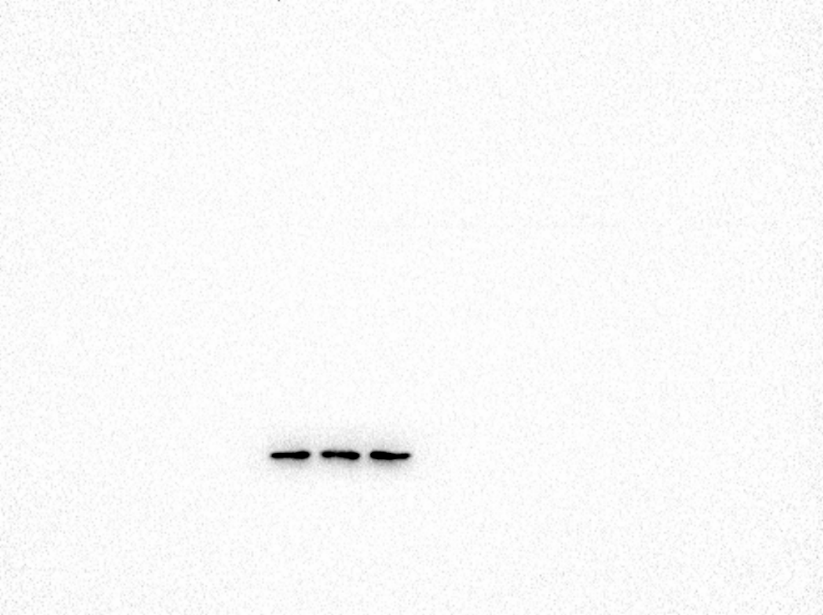


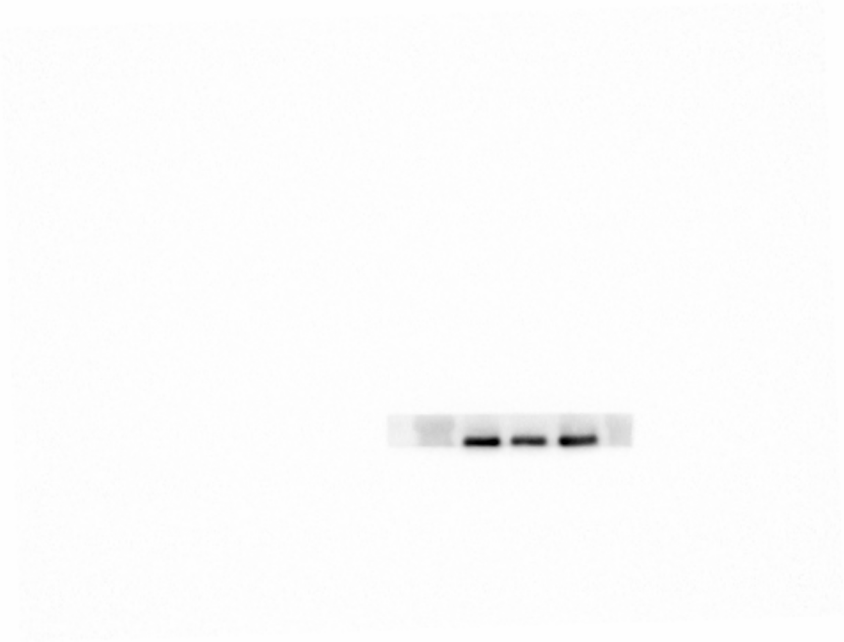

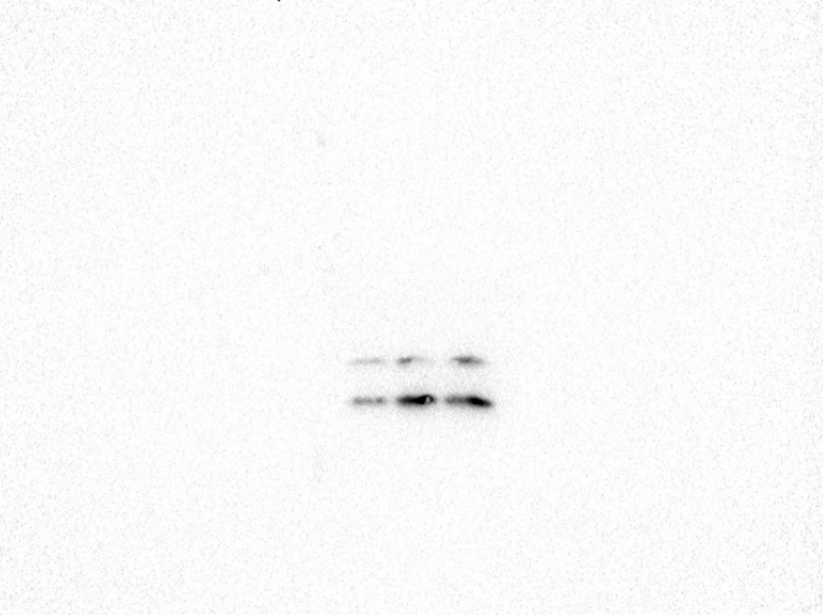

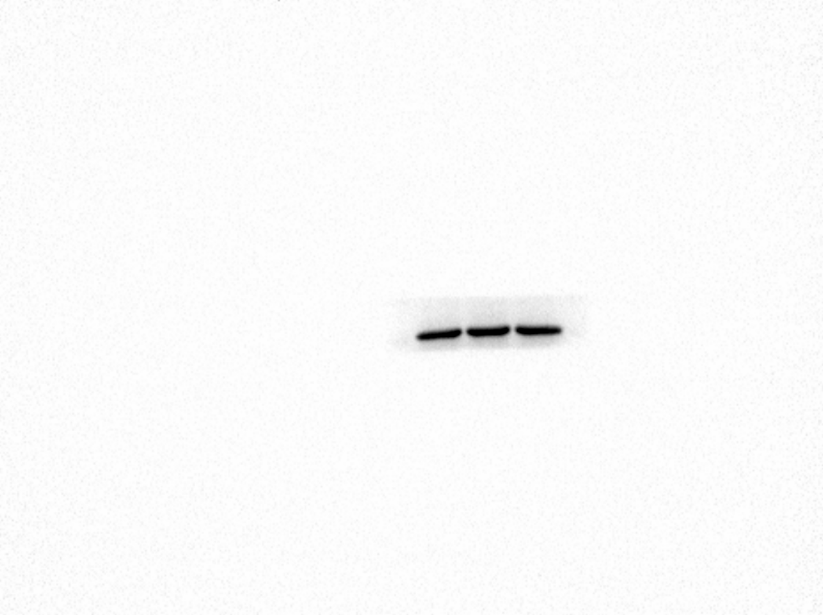


**Figure 4**


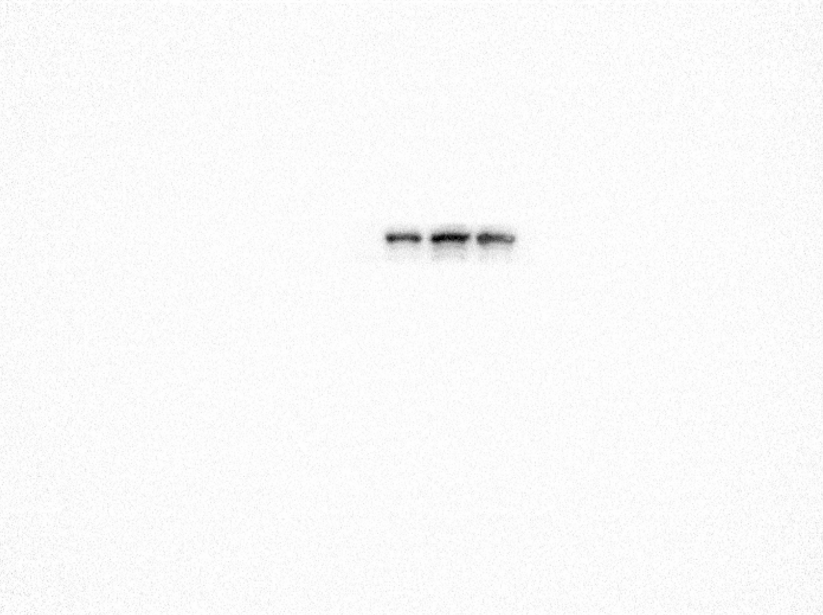

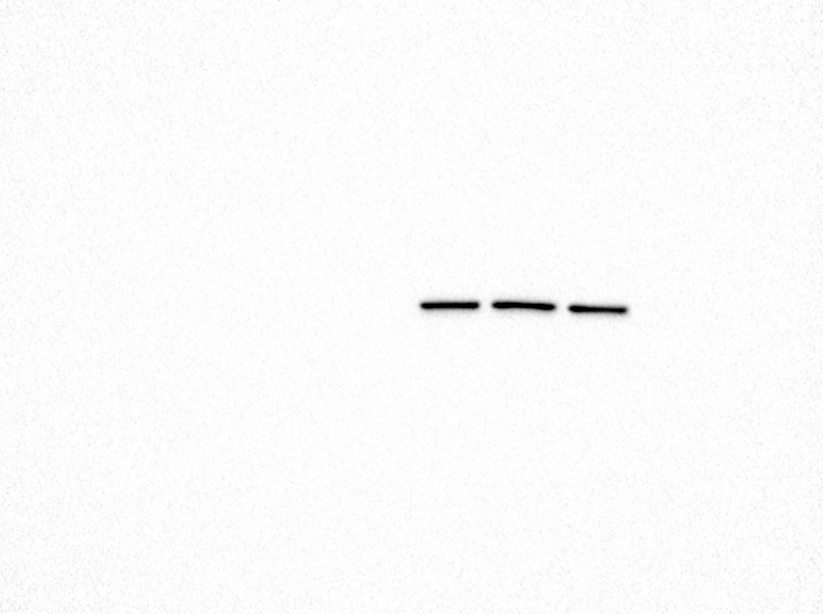


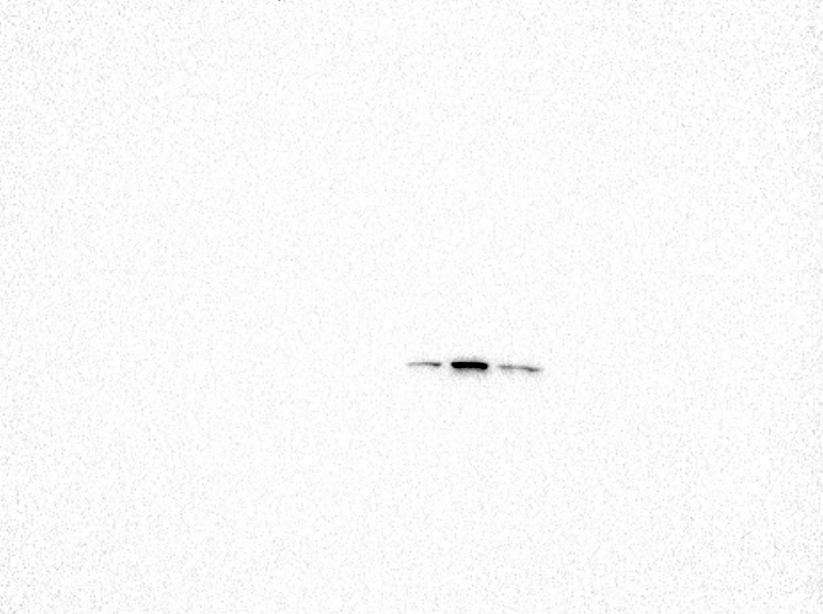

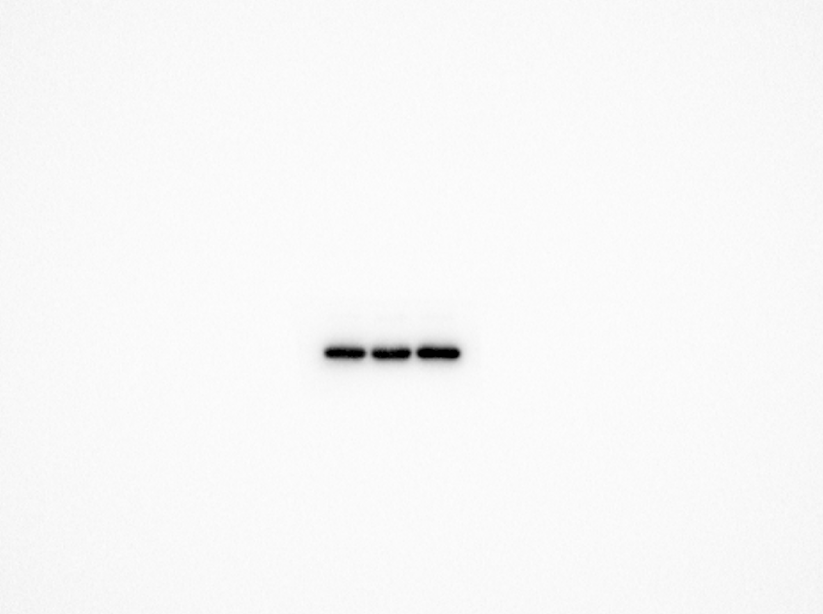


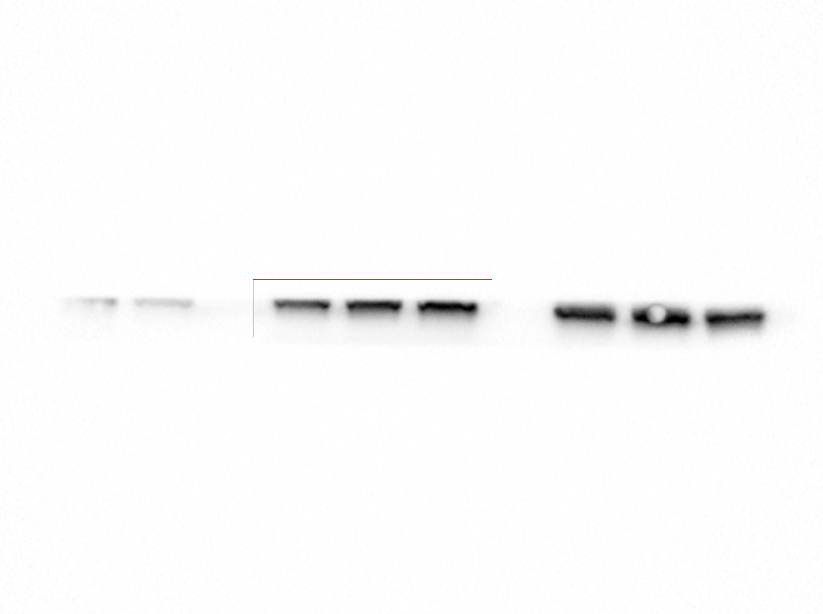

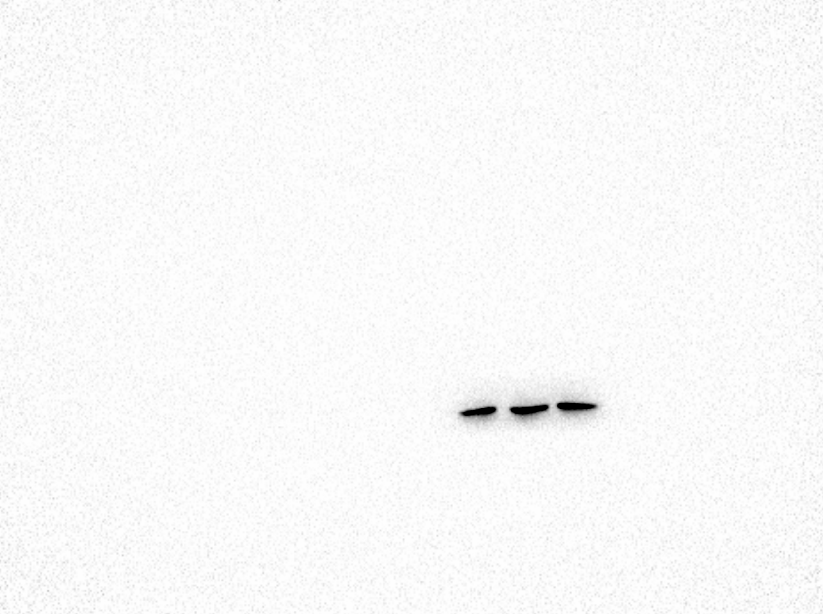


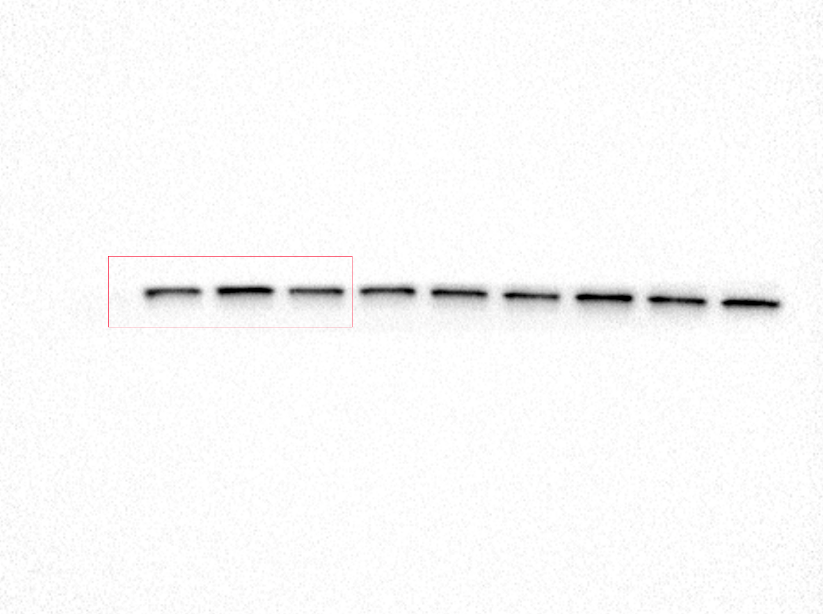

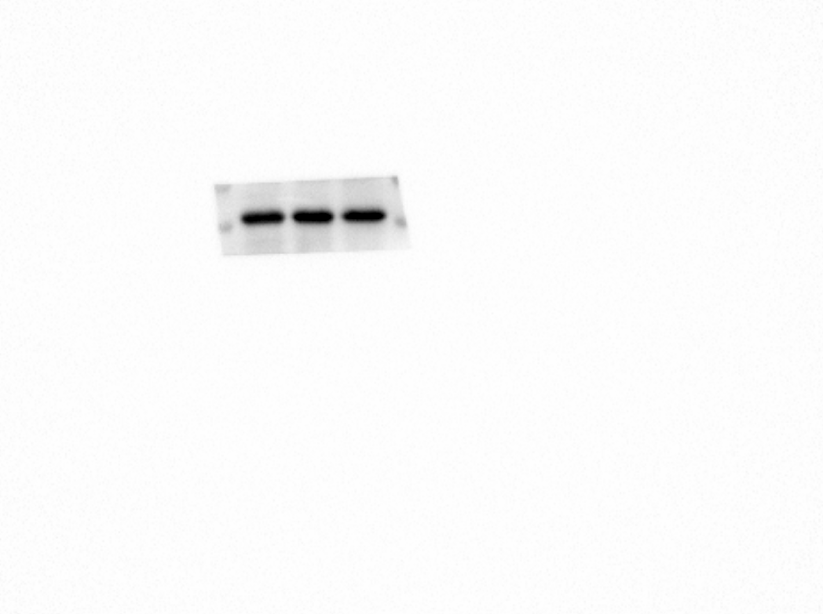


**Figure 6**


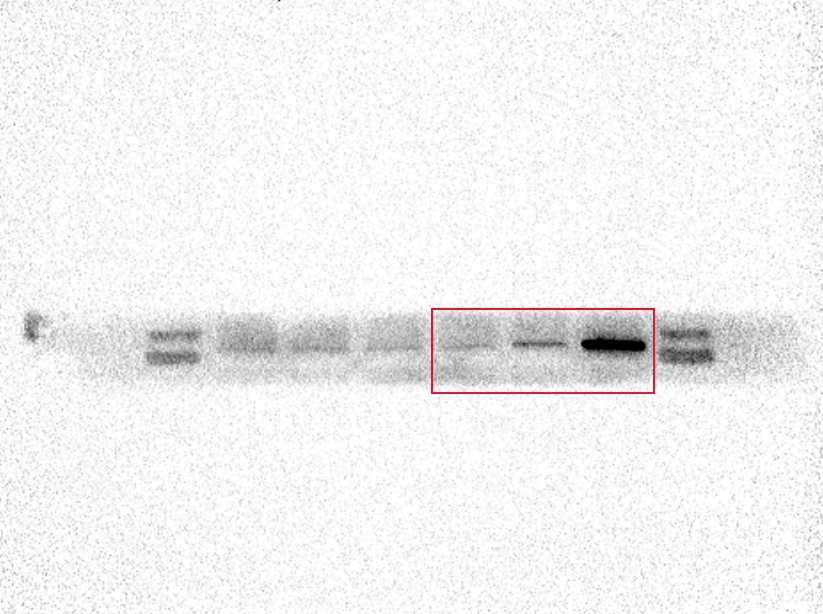

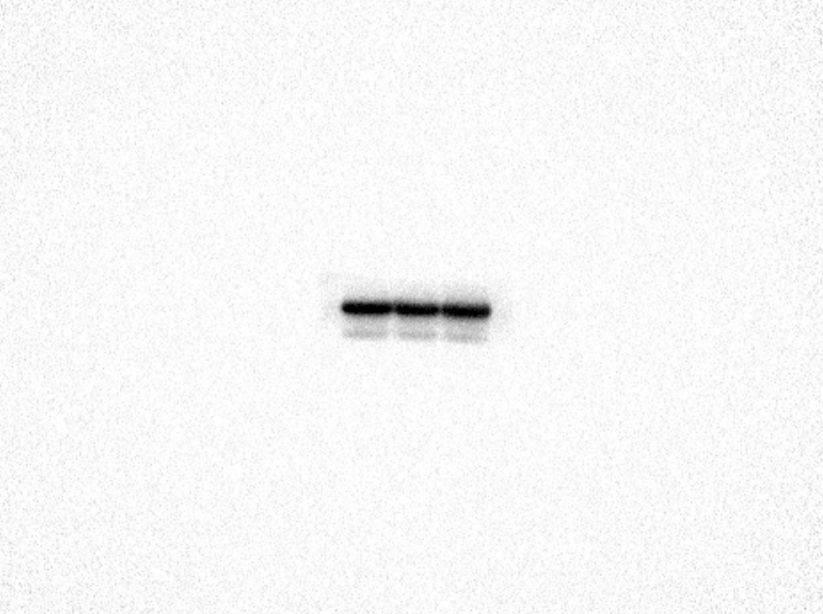


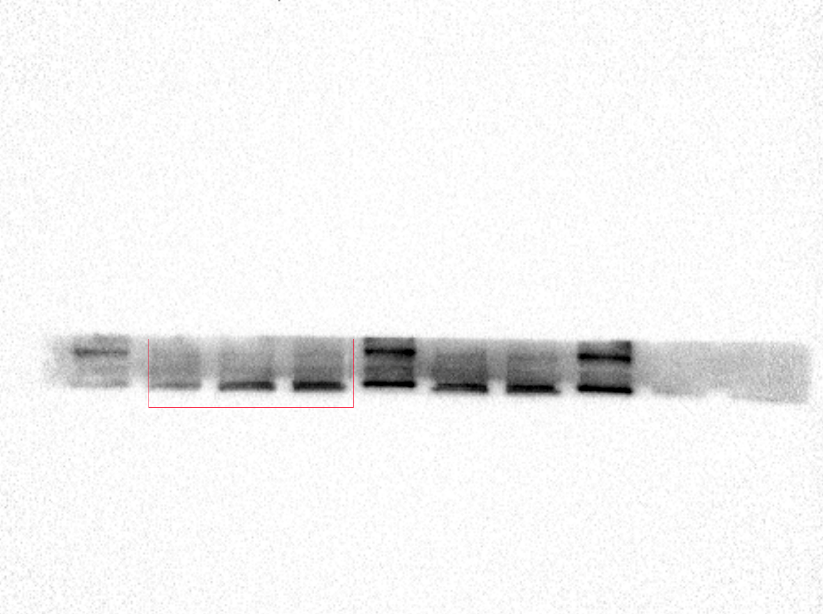

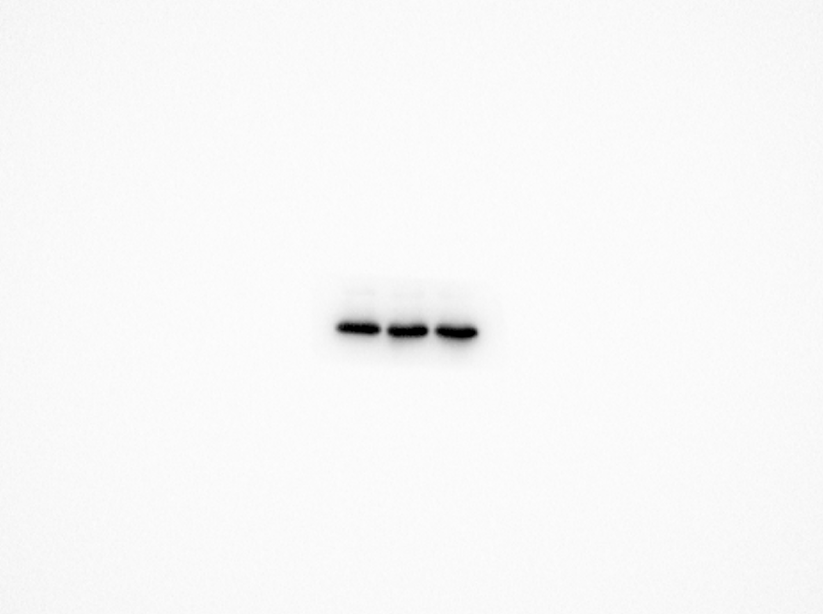


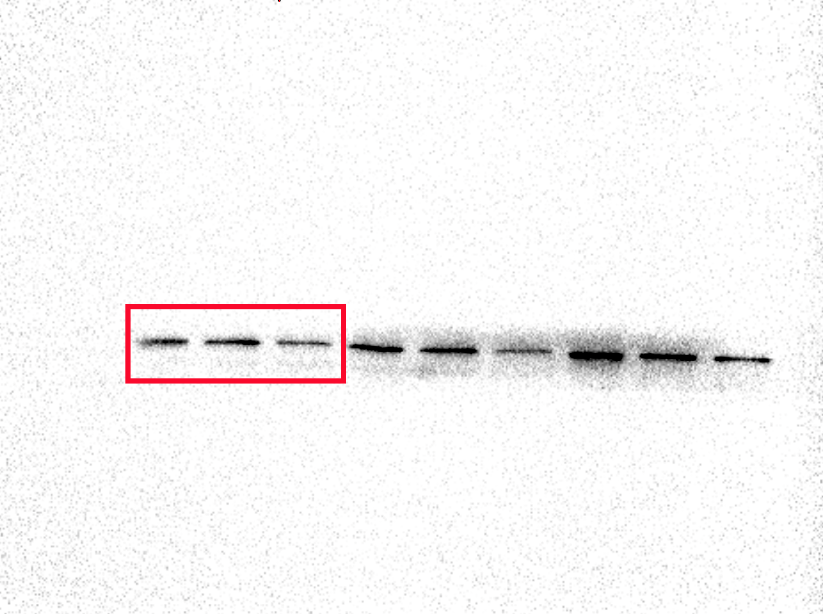

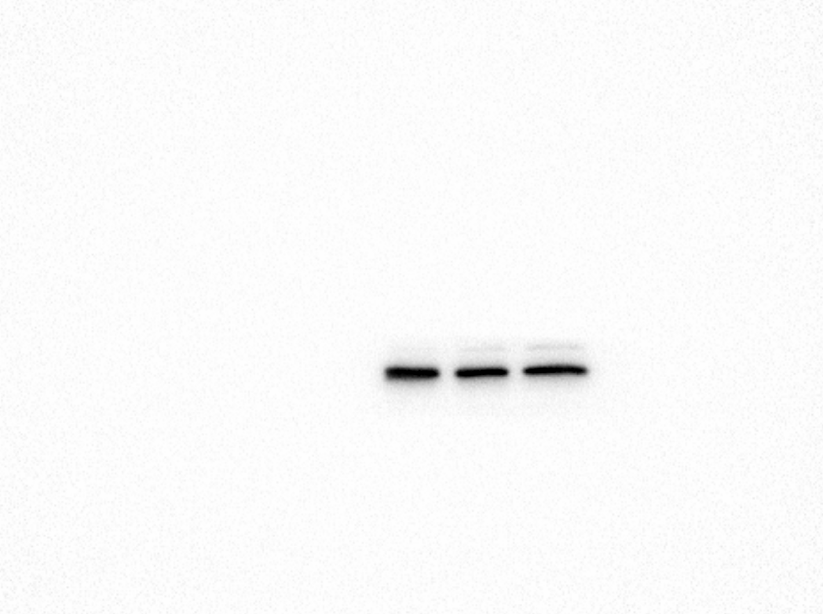


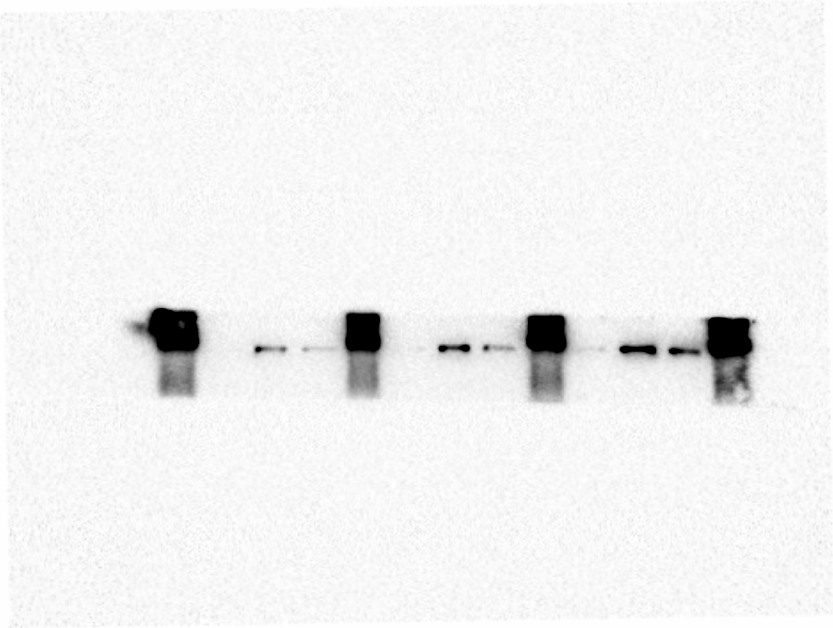


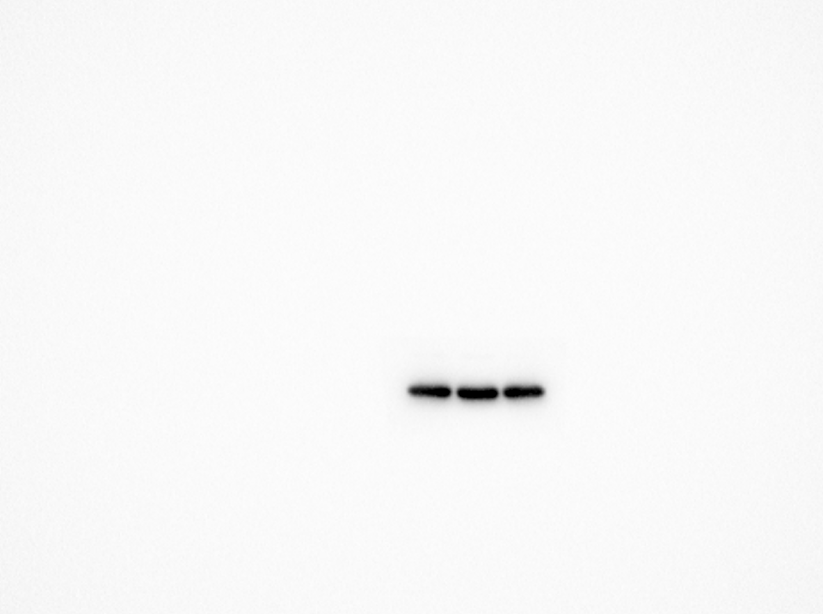


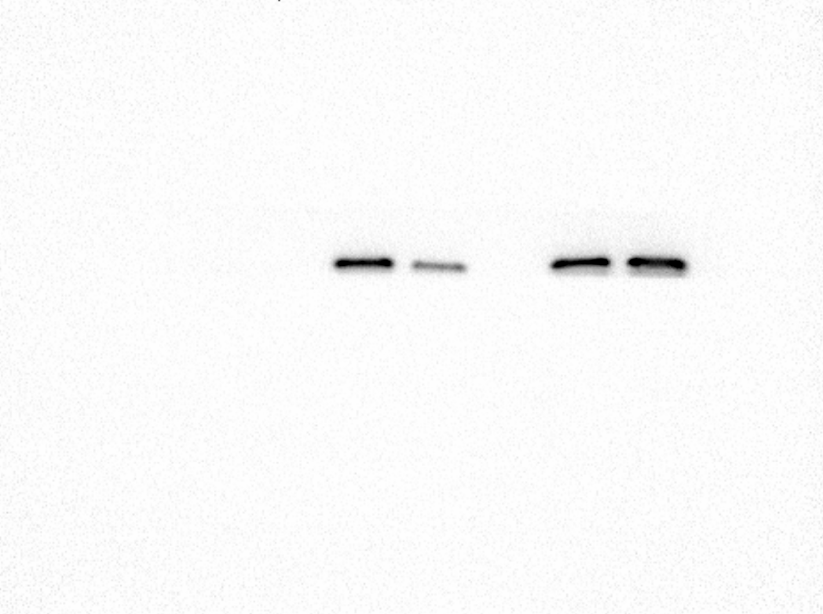


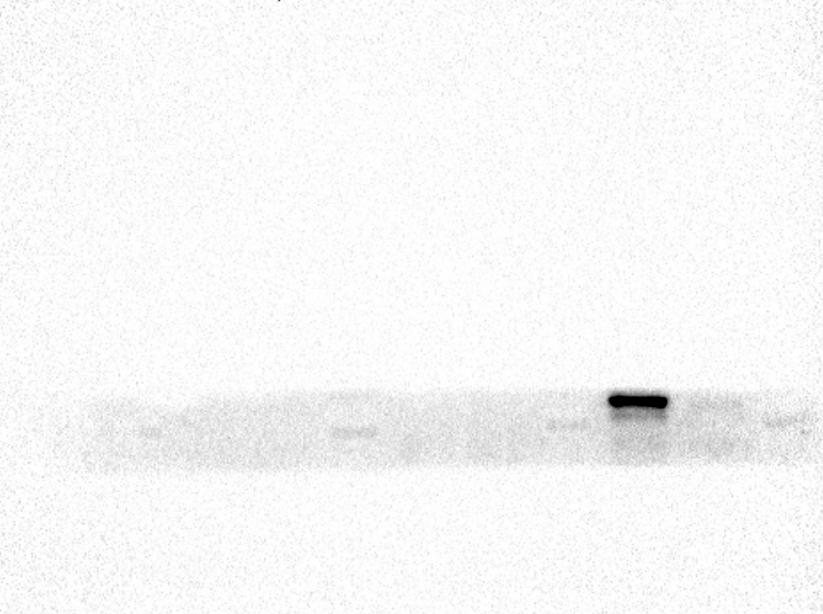

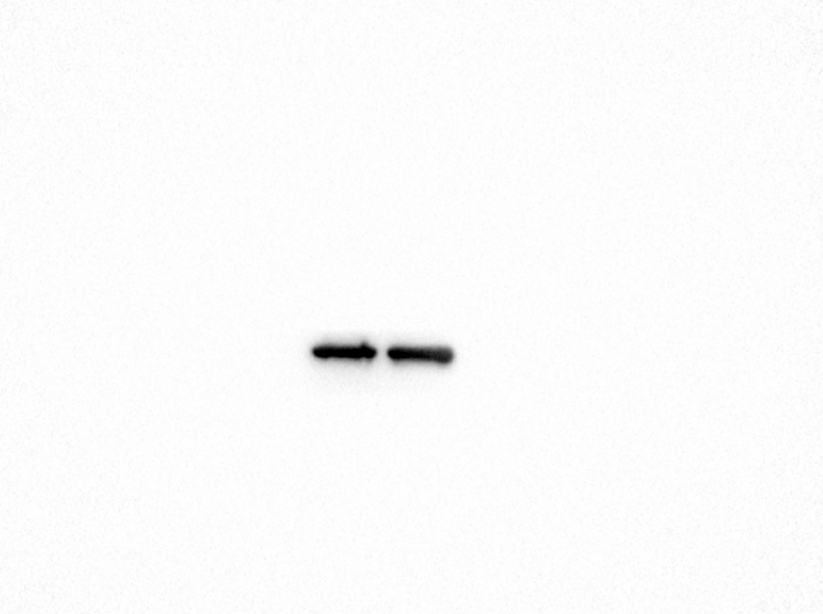


**Figure 7**


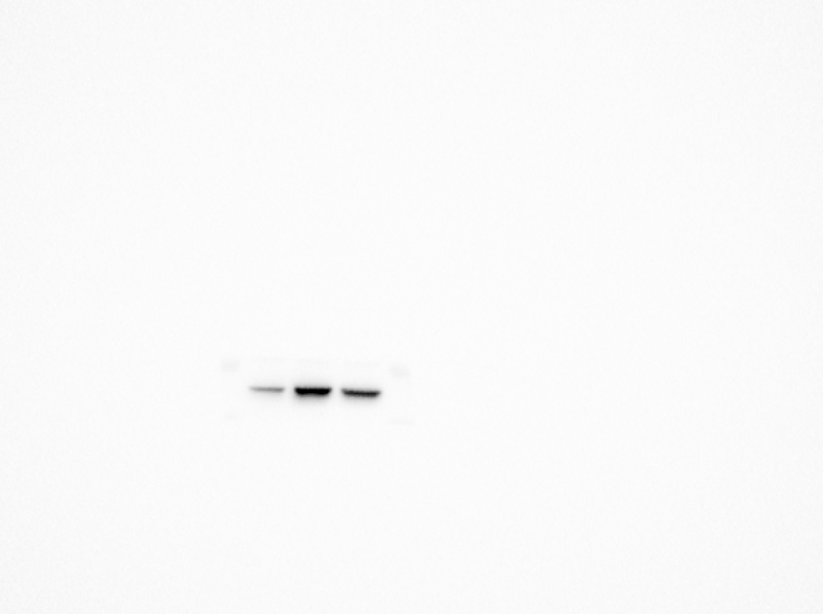


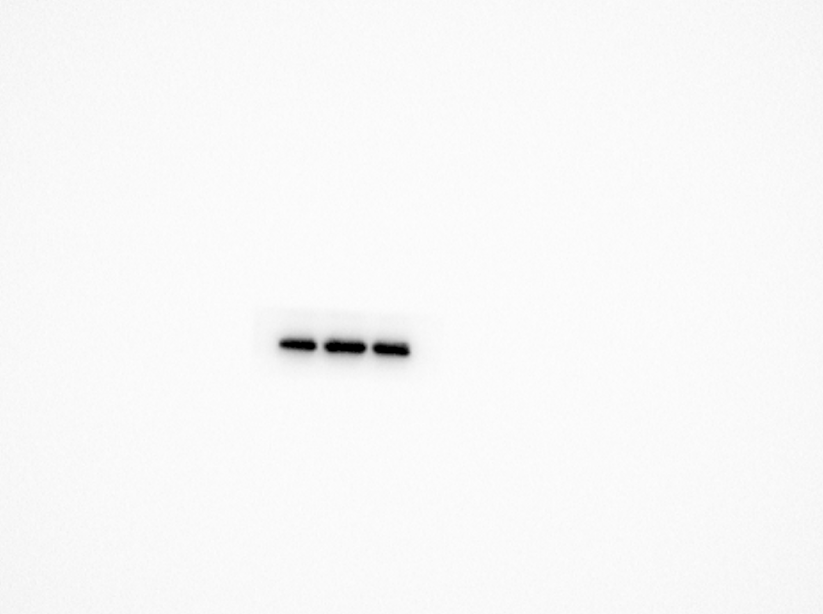


**Figure s7**


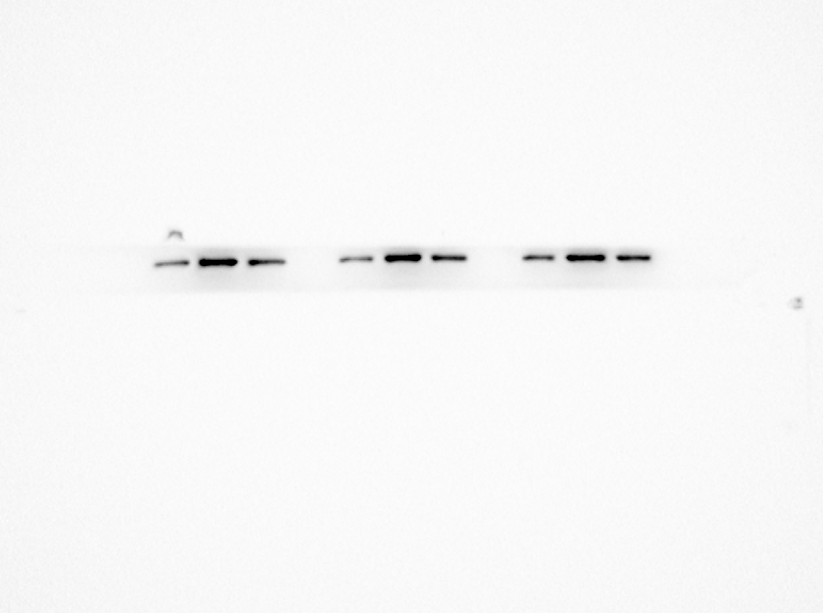


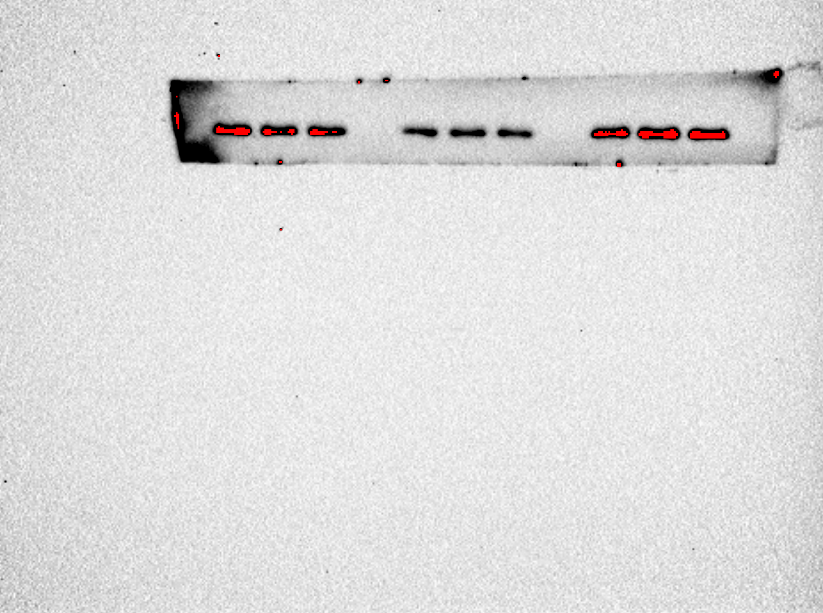

Supplement: Supplementary file 9 — original western blot [file 41419_2025_7576_MOESM9_ESM.docx]
